# Innexin expression and localization in the *Drosophila* antenna indicate gap junction or hemichannel involvement in antennal chemosensory sensilla

**DOI:** 10.1007/s00441-024-03909-3

**Published:** 2024-08-23

**Authors:** Sinisa Prelic, Ian W. Keesey, Sofia Lavista-Llanos, Bill S. Hansson, Dieter Wicher

**Affiliations:** Dept. Evolutionary Neuroethology, Max Planck Institute for Chemical Ecology, Jena, Germany

**Keywords:** Insect, Olfaction, Innexon, OSN, Support cell

## Abstract

Odor detection in insects is largely mediated by structures on antennae called sensilla, which feature a strongly conserved architecture and repertoire of olfactory sensory neurons (OSNs) and various support cell types. In *Drosophila*, OSNs are tightly apposed to supporting cells, whose connection with neurons and functional roles in odor detection remain unclear. Coupling mechanisms between these neuronal and non-neuronal cell types have been suggested based on morphological observations, concomitant physiological activity during odor stimulation, and known interactions that occur in other chemosensory systems. For instance, it is not known whether cell–cell coupling via gap junctions between OSNs and neighboring cells exists, or whether hemichannels interconnect cellular and extracellular sensillum compartments. Here, we show that innexins, which form hemichannels and gap junctions in invertebrates, are abundantly expressed in adult drosophilid antennae. By surveying antennal transcriptomes and performing various immunohistochemical stainings in antennal tissues, we discover innexin-specific patterns of expression and localization, with a majority of innexins strongly localizing to glial and non-neuronal cells, likely support and epithelial cells. Finally, by injecting gap junction-permeable dye into a pre-identified sensillum, we observe no dye coupling between neuronal and non-neuronal cells. Together with evidence of non-neuronal innexin localization, we conclude that innexins likely do not conjoin neurons to support cells, but that junctions and hemichannels may instead couple support cells among each other or to their shared sensillum lymph to achieve synchronous activity. We discuss how coupling of sensillum microenvironments or compartments may potentially contribute to facilitate chemosensory functions of odor sensing and sensillum homeostasis.

**Supplementary information:**

The online version contains supplementary material available at 10.1007/s00441-024-03909-3.

## Introduction

Insects detect chemical cues using conserved sensory structures called sensilla. Sensilla are hair-like cuticular protrusions found abundantly in all insect species, across many body parts and appendages, and are involved in the survival-critical sensory modalities of both gustation and olfaction (Krieger and Breer 1999; Vosshall 2000; Ache and Young 2005). In *Drosophila*, sensilla occur most densely and numerously on antennae, which are a pair of versatile sensory organs frontally situated on the head that combine multiple Aristotelian senses within two antennal segments. The second antennal segment, or pedicel, hosts the Johnston’s organ, an organ composed of radially arranged scolopidia detecting motion transmitted throughout the antenna for purposes of gravitaxis, audition, and proprioception (Kamikouchi et al. 2009; Boekhoff-Falk and Eberl 2014). The anatomically larger third antennal segment, or funiculus, hosts a diverse set of well-explored chemosensory sensilla which have been intensely atlased to better understand the chemical ecology of flies and the underpinnings of odor reception and transduction at the molecular and sensory neuronal levels. For instance, the combinatorial expression of different semiochemical-specific receptors across many classes of olfactory sensory neurons (OSNs) has been thoroughly investigated and described over recent decades (e.g., Laissue and Vosshall 2008; Gomez-Diaz et al. 2018; Montell 2021).

Notably, all sensilla, including those found on the antennae, are composed of an aqueous, extracellular compartment called the sensillum lymph, and a repertoire of sensory neurons and non-neuronal support cells arranged in a highly conserved morphological architecture. A typical sensillum involved in detecting odors is invariably composed of 1-or-few OSNs dendritically innervating the lymph and protruding sensillum shaft, surrounded by a layered triad of support cells termed thecogen (sheath), trichogen (shaft), and tormogen (socket) support cells (Shanbhag et al. 1999, 2000; Nava Gonzales et al. 2021; Prelic et al. 2022). The arrangement is highly similar across all insect sensilla: innermost, the thecogen cell tightly envelops the somata and inner dendrites of OSNs, while the tormogen and trichogen cells flank the sensillum compartment to enclose the inner cells and lymph with numerous sealing septate junctions, functionally isolating the sensillum organ from neighboring sensillum units (Steinbrecht 1996; Chai et al. 2019). This cytological organization is strongly conserved among all types of chemosensory sensilla, and also between chemosensory sensilla and mechanosensory organs such as scolopidia, as these sensory structures share structural and developmental homology (Keil and Steinbrecht 1984; Foelix et al. 1989; Yack 2004). The cellular heterogeneity and associated microenvironments of sensilla thus underlie the extraordinary sensory abilities of insects.

Though sensory mechanisms of *Drosophila* chemosensory neurons have been largely unraveled, the functional roles of the remaining set of non-neuronal support cells remain strikingly unclear, especially with regard to how they modulate the specific and sensitive processes of chemosensory transduction, as well as their role in sensory systemic properties such as maintaining lymph composition, and sensitization or adaptation to repeated presentation of chemical cues (Larter et al. 2016; Schmidt and Benton 2020). In insects, antennal support cells remain difficult to isolate, manipulate, and are poorly characterized at the molecular level, thereby making it difficult to experimentally assess their role in chemical cue detection (Schmidt and Benton 2020). To this day, heterogeneity and variability in biological function of different support cell subtypes also remains largely unexplored (Prelic et al. 2022; Scalzotto et al. 2022).

Nevertheless, there exist insights from sensory systems of disparate organisms which suggest perineuronal support cells play crucial roles in modulating and potentiating neuron activity. One mode of functional interaction between cells is through intercellular coupling via gap junctions. Gap junctions are direct cell-to-cell contacts, wherein partnered cells are directly interconnected through specialized gap junction channels, or “bridges,” which allow direct communication and exchange of molecules and signals between associated cells (Goodenough and Paul 2009; Pereda 2014). Gap junctions have been functionally implicated in many sensory systems and their constituent support cells over the years. For instance, gap junctions interconnect varieties of non-neuronal support cells in the rat cochlea (Kikuchi et al. 1995), which includes structural and electrical coupling of interior and exterior supporting cells of the organ of Corti with gap junctions (Heinrich and Helling 2012), and where important sensory functions like auditory amplification are actively dependent on this junctioning phenomenon between supporting cells (Zhu et al. 2013). The regulation of the cochlear gap junction system, or dysfunction thereof, has been implicated in sensory hearing disorders (Heinrich and Helling 2012; Zhu et al. 2013).

In insects and closely related invertebrate clades, it has long been speculated that cells within chemosensory sensilla may also be directly connected via such gap junctions (Steinbrecht 1980). From electron microscopic observations, hints of gap junctional connections between closely apposed plasma membranes have been tentatively noted and suggested to exist as means for intercellular communication (Foelix et al. 1975, 1989; Gaffal 1976; Keil 1978; Steinbrecht 1980). Since then, the genes and proteins that constitute gap junctions in invertebrates have been discovered (Phelan et al. 1998a) and in part functionally characterized (Phelan et al. 1998b; Stebbings et al. 2002; Hughes 2014). Members of the innexin protein family were shown to compose homo- and heteromeric gap junctions, forming bidirectional connections between cytoplasms of cells, as well as half-channels (hemichannels) that constitute open pores across plasma membranes into the extracellular milieu (Phelan et al. 1998a; Blagburn et al. 1999).

In the context of sensory systems, gap junctions have been found in the retina, wherein many neuron types exhibit electrical synapses that are understood to undergo dynamic regulation and play roles in both transmission and processing of visual information at the earliest sensory levels (Bloomfield and Völgyi 2009). This phenomenon is also present in insects, as evidenced by studies on the retina and lamina cartridges of the *Drosophila* compound eye, which are underpinned by innexin proteins (Meinertzhagen and O’Neil 1991; Shimohigashi and Meinertzhagen 1998; Curtin et al. 2002; Stebbings et al. 2002). Here, some innexins have been shown to compose gap junctions between photoreceptors; examples of coupling between axons and non-neuronal channels have also been described in adult specimens, but are more sparse and have been noted to possibly contribute to functions unlike those between neuronal cells alone (Curtin et al. 2002)*.* In the *Drosophila* auditory system, auditory sensory cells were similarly shown to couple with the giant fiber in *Drosophila* via innexin gap junctions (Jezzini et al. 2018). Though now evident that sensory neurons of the visual and auditory periphery interface with downstream neurons via electrical synapses, neither gap junctions, innexins, nor electrical synapses have been investigated in OSNs at the insect olfactory periphery. In addition to this, gap junctions and innexins appear absent in the axonal termini of OSNs, which innervate the antennal lobe in the insect brain (Yaksi and Wilson 2010; Wilson 2013; Das et al. 2017). This leaves an unexplored possibility that any putative gap junctions in OSNs may function exclusively in the insect antenna alone, where they have not yet been investigated. Given that the set of innexin genes themselves are undercharacterized in most insect tissues (Hughes 2014; Güiza et al. 2018), as well as a relative lack of insight into the role or presence of gap junctions or hemichannels within sensory systems (Skerrett and Williams 2017), many questions remain.

Though olfactory transduction is entirely dependent on the maintenance of a transepithelial voltage at the sensillum, itself dependent on ion homeostasis of the sensillum lymph, speculations about electrical coupling between antennal support cells which underpin the electrical properties of the sensillum and the cycling of ions have yet to be resolved (Küppers and Thurm 1979; Thurm and Kuppers 1980). Likewise, short-range lateral inhibition between OSNs residing in the same sensillum is commonly observed and reported (Su et al. 2012; Zhang et al. 2019; Ng et al. 2020), but the mechanisms of inhibition are unclear and contributions of hemichannels or gap junctions potentially located between the tightly apposed support cells and/or neurons have not been experimentally addressed nor definitively ruled out (Su et al. 2012; Zhang et al. 2019; Ng et al. 2020; Pannunzi and Nowotny 2021; Wu et al. 2022). Curiously, few hints implicate involvement of gap junctions in chemosensory sensilla. First, a recent study has shown innexin activity in chemosensory sensilla found on *Drosophila* wings, demonstrating impairment in Ca^2+^ flux in flies exhibiting genetic silencing of a pre-identified innexin of abundant expression on the wing (Raad and Robichon 2019). Here, a functional role of innexins beyond that of development (i.e., sensillum morphogenesis)—namely a functional role in mature sensilla—was tentatively suggested (Raad and Robichon 2019). Second, an electrophysiological study in bumblebees employing gap junction blockers showed that peripheral gustatory neuron bursting (a characteristic temporal pattern of spiking) is facilitated by gap junctions, which permits resistance to sensory adaptation already at the sensory neuron level (Miriyala et al. 2018). In particular, this demonstration concerns large, *A*-type, galeal chemosensory sensilla of the bee proboscis which host two sensory neurons (Miriyala et al. 2018), a system similar to that of antennal sensilla. Together, these insights further call into need an elementary investigation characterizing innexins in insect antennae.

In attempt to address this knowledge gap, in this descriptive study we investigate the expression and localization of innexins within the *Drosophila* antenna on both RNA and protein levels. The approach consists of bioinformatic data mining of a wide variety of relevant antennal transcriptomes to show evidence of the expression of a subset of innexins in cells of the *Drosophila* antenna, with consideration of enrichment and depletion of specific innexin genes in antennal cell types, olfactory subsystems, and sensillum types. Subsequently, we employ optical imaging in tandem with immunohistochemical stainings of innexins, for purposes of demonstrating the presence and localization of these hemichannel- or gap junction-forming genes in the antenna, with special focus on the third (largely olfactory) segment of the antenna. Last, by way of employing gap junction-permeable tracer dyes in a chemosensory sensillum case study, we identify no dye coupling between neurons and perineuronal support cells. In light of these observations, we finally present and discuss potential roles and functions of antennal innexins, and correspondingly, gap junctions and hemichannels, within the complex multicellular compartments of insect chemosensory sensilla.

## Materials and methods

### Transcriptomics and expression visualization

Transcriptomes from five independent RNA-seq studies sampling antennal tissue were surveyed for antennal gene expression. We consulted the following antennal datasets: RNA-seq pooling 300 mixed-sex flies, 5–12-days-old post-eclosion, for two *D. melanogaster* genotypes, wildtype Canton-S flies and homozygous *atonal* (*ato*) mutants (Menuz et al. 2014); RNA-seq pooling 300–400 antennae each from mixed-sex flies of 3–5-day-old age and of wildtype Canton-S strain and homozygous *amos* mutant fly line (Mohapatra and Menuz 2019). These two studies were selected for purposes of comparing across contexts of expression concerning IR and OR olfactory subsystem deficiency. Additionally, an RNA-seq dataset sampling 1200 antennae pairs for each sex in Canton-S flies aged > 1-day post-eclosion was consulted to compare between fly sexes (Shiao et al. 2013), and an RNA-seq dataset sampling antennae of 6 *Drosophila* spp. wherein 300 mixed-sex antennae for each species in flies aged 7–10-days post-eclosion were sampled (Pan et al. 2017). Gene expression abundance of all eight *Drosophila* innexins were plotted against antennal and/or cell type-demarcating (*Orco*, *elav*, *repo*, *nompA*, *Su(H)*, *Snmp1*, *Snmp2*) and common housekeeping (*Act5C*, *Gapdh1*, *Cam*, *eEF1β*) reference genes for relative overview. Genes were selected based on broad function and/or representation of variety of cell types, including markers for non-neuronal cells of the antenna, and for robustness, a variety of housekeeping genes involved in pan-cellular, basic processes involving ubiquitous cytoskeletal, metabolic, physiological, and enzymatic functions (Lü et al. 2018). Error bars indicate standard error of the mean (SEM) where multiple replicates were performed or available. Whole transcriptome expression curves were plotted by ordering positively detected genes of non-zero expression within each corresponding condition or grouping, colored by percentile rank of every gene among all detected genes, calculated in both R and Microsoft Excel (PERCENTRANK function). Where appearing, antennal innexin genes were labeled with text centered on their datapoint, and displaced vertically away from their point on the curve for readability. For the RNA-seq dataset concerned with cross-drosophilid comparison, *D. melanogaster* was compared against expression means for the remaining five non-*D. melanogaster* species to gauge the difference between model and non-model drosophilid innexin expression abundance.

All single-cell transcriptomes used were obtained from the Fly Cell Atlas (Li et al. 2022a). The datasets used come from antennal tissue-specific scRNA-seq with cells isolated either using a microfluidic droplet-based cell-capture 10X methodology or plate-based SMART-seq2 methodology (Li et al. 2017, 2022a; McLaughlin et al. 2021). Both 10X datasets originating from “stringent” and “relaxed” datasets were data mined in parallel with the SMART-seq2-derived dataset. Cell group classifications are based on the groupings “*annotation_broad*” and “*annotation*” for all 10X antennal datasets, and “*transf_annotation*” for the SMART-seq2-derived antennal dataset. Datasets merging 10X- and SMART-seq2-sourced transcriptomes were not considered to avoid confoundment. The single cell transcriptomic visualization platform SCope (Davie et al. 2018) was used for visualizing tSNE plots where gene expression (corrected transcript count) of detectable innexins and major molecular cell–type markers (*Orco*, *sv*) is visualized by color on a min–max basis using default settings.

### Differential expression analysis in antennal scRNA-seq datasets

All differential expression analysis performed comparing cell group classifications on specific gene expression used non-parametric Wilcoxon rank-sum statistical testing (via Seurat) with default parametrization using the online Automated Single-cell Analysis Pipeline (ASAP) portal at asap.epfl.ch (Gardeux et al. 2017). Wilcoxon rank-sum tests were chosen as suitable and false positive-conservative tests to detect significant enrichment or depletion within cell groupings, as the test has no requirements on gene expression distribution and an informative null hypothesis to test against regardless of distribution skewness (Li et al. 2022b). A statistical significance threshold of false detection rate (FDR) adjusted *p*-value (also called *q*-value) of < 0.05 was used to differentiate significantly up- or downregulated genes from insignificantly differentially expressed genes. Parametrization on ASAP was used as follows: *minimum % of cells with gene* > *0* = 0.1 (10%); *false detection rate limit* = 0.05; *min%diff* = NULL; *max cells per group* = NULL; *foldchange cutoff* = 1.3; max cells per group = NULL. *Foldchange cutoff* was set to 1.3 or 2.0 (i.e., approx. 2.5-fold and fourfold difference in expression) to differentiate between weak and strong detection of gene transcript enrichment or depletion between cell groups. For bubble plot figures, only significant differential expressions are plotted (i.e., blanks denote statistically insignificant cell group–specific expressions). Where significant differential expression is found, a bubble is drawn, colored by log2-scaled gene expression foldchange (depletion tending red, enrichment tending green) and sized based on foldchange magnitude (absolute value) for ease of visualization. Within the visualization, cell classifications analyzed were ordered and grouped by classification type (e.g., broad, narrow, custom) and by attributable cell type (e.g., non-neuronal cells, IR subsystem OSNs). For variant bubble plots appearing in supplementary figures, differentially expressed genes detected at *foldchange cutoff* > 1.3 but < 2.0 were rendered circled, smaller and transparently shaded, while genes detected at > 2.0 were rendered large and opaque (see legend). A variety of queried antennal datasets from the Fly Cell Atlas were also selected for comparative robustness. We looked at data originating from different single-cell isolation methods (10X and SMART-seq2), within datasets generated from raw data by different data processing pipelines (*stringent* vs. *relaxed* datasets), and across different kinds of annotations of cell type (cell “groupings” or “classifications”), which are categorized manually by crowd annotation and/or through clustering (e.g., “*annotation_broad*” discriminates broadly between general cell type; *“annotation”* discriminates between cell subtype, especially within the sensory neuron class).

All visualizations were created using custom scripts written using the ggplot2 package of R in RStudio, and are made available in the public data repository. Gene transcripts are italicized and references to protein counterparts are unitalicized in line with FlyBase guidelines on nomenclature. Gene and protein nomenclature is presented based on FlyBase’s (flybase.org) gene symbol and name; in studies incorporating “outdated” names or Flybase ID, gene labels were converted to gene FlyBase symbol and name (e.g., where applicable, Or83b was renamed to Orco for cross-study consistency). As an exception, *eEF1β* is referred to by its synonym *Ef1beta*.

### Fly stocks and rearing

Transgenic *D. melanogaster* lines carrying Gal4/UAS elements were obtained, balanced, reared, and crossed as done previously (Prelic et al. 2022). All fly lines were obtained from the Bloomington Drosophila Stock Center (BDSC, Bloomington, Indiana) except where noted otherwise. *D. melanogaster* strains used are as follows (including BDSC stock ID and original sources in parentheses): Orco-Gal4 (26818 and (Wang et al. 2003)), nompA-Gal4 and ASE5-Gal4 (Craig Montell, University of California, Santa Barbara, and (Barolo et al. 2000)), Or67d-Gal4 (Kurtovic et al. 2007), Or47b-Gal4 (9984 and (Vosshall et al. 2000), and UAS-mCD8-GFP (5137 and (Lee and Luo 1999)). All *D. melanogaster* stocks were maintained on conventional cornmeal agar medium (500 ml recipe: 59 g treacle, 5.4 g Brewer’s yeast, 101 ml hot water, 2.1 g agar, 135 ml cold water, 47 g polenta, fill up with 135 ml hot water, flush out with 34 ml hot water, 54 ml cold water, 1.2 ml propionic acid, 1.65 ml NIPAGIN 30%) under a 12 h/12 h light/dark cycle. *D. suzukii* flies were obtained from the National Drosophila Species Stock Center (NDSSC) at Cornell University (Ithaca, New York), stock number/reference specimen: 14023–0311.01. These stocks were maintained on a standard diet of conventional cornmeal agar medium supplemented with freshly crushed blueberries at 22 °C and 40% humidity under a 12 h/12 h light/dark cycle, as described previously (Keesey et al. 2019). The study was conducted in Germany where research on invertebrates requires no animal research committee approval; the fly laboratory meets all requirements of the Thuringian State Office for Consumer Protection (verbraucherschutz.thueringen.de).

### Immunohistochemistry and optical imaging of whole antennae and antennal sections

Antibodies and chemicals used, including information pertaining to identity, source, epitope, and dilution, are listed in Table 1. For all immunostaining procedures, antennae were dissected under magnification by excision using a fine needle and placed into excess *Drosophila* Ringer solution (5 mM HEPES; 130 mM NaCl; 5 mM KCl; 2 mM MgCl2; 2 mM CaCl2; 36 mM sucrose, aqueous), equilibrated prior to pH = 7.30 and room temperature (RT), to maintain physiological conditions and prevent desiccation. Antennae were then fixed or cryosectioned onto microscope slides for immunohistochemical staining.

**Table 1:** List of antibodies and tracer chemicals used

**Primary antibodies (pAb)**					
**Epitope**	**pAb**	**Source**	**Note**	**Experiment**	**Dilution**
Heterologous GFP	Ch anti-GFP	Invitrogen	polyclonal	Whole and sectioned antennae	1:500
ogre	Rb anti-ogre	Reinhard Bauer	-	Whole and sectioned antennae	1:50
Inx2	Rb anti-Inx2	Reinhard Bauer	-	Whole ad sectioned antennae	1:100
Inx3	Rb anti-Inx3	Reinhard Bauer	-	Whole and sectioned antennae	1:50
shakB	Rb anti-shakB	Georg Ammer	GenScript antibody	shakB staining in whole antennae	1:2000
shakB	Rb anti-shakB	Georg Ammer	Serum antibody	Not shown (no staining evident in whole antennal mounts)	1:800
zpg	Rb anti-zpg	Guy Tanentzapf	Peptide antibody	Sectioned antennae	1:50
Inx2	Gp anti-Inx2	Guy Tanentzapf	Peptide antibody	Immunocontrol for sectioned antennae	1:50
**Secondary antibodies (sAb) or tracer detection**					
**Epitope**	**sAb**	**Source (conjugate)**	**Note**	**Experiment**	**Dilution**
Chicken pAb	Gt anti-Ch::A488	Invitrogen (Alexa Fluor 488)	-	Colocalizing cell type-specific GFP expression with various innexin immunostainings	1:250
Rabbit pAb	Gt anti-Rb::A546	Invitrogen (Alexa Fluor 546)	-	Innexin immunostaining and immunocontrols	1:250
(neuro)Biotin	Streptavidin::A555	Invitrogen (streptavidin-Alexa Fluor 555 conjugate)	S21381	Neurobiotin detection following SSR-guided single sensillum backfilling	1:1000
**Chemicals**					
**Tracer**	**Size**	**Source**	**Note**	**Experiment**	**Dilution**
Neurobiotin	MW: 322.8 Da	VectorLabs	SP-1120	SSR-guided single sensillum backfilling and whole antennal reverse backfilling	1–2% dye solution

*Ch* chicken, *Rb* rabbit, *Gt* goat, *Gp* guinea pig

Immunostaining of whole antennae involved fixation of 30–100 intact antennae per staining on ice in a shaker for 2 h in 4% paraformaldehyde (PFA) in phosphate-buffered saline (PBS) supplemented with 0.1% Triton X-100 (Sigma-Aldrich, Darmstadt, Germany), followed by four washes for 15 min in PBS with 0.1% Triton X-100 (PT solution). Whole antennal samples were then blocked for 1 h at RT in 5% normal goat serum (NGS) in PT solution (PTS solution). Following, blocking solution was exchanged for primary antibody solution, which was prepared by diluting antibodies as detailed in Table 1 in PTS solution, and incubated for 2 days at 4 °C. Subsequently, the sample was washed four times in PT solution for 15 min per wash, blocked again for 2 h in PTS solution at RT, and incubated with secondary antibodies prepared in PTS solution overnight at 4 °C in the dark under mild rocking using a shaker. Last, the samples were washed three times in PT solution for 15 min per wash, and whole antennae were mounted in Vectashield (Vector Laboratories, Newark, CA, USA) for imaging whole antennal samples.

Antennal sections were prepared by depositing 30–100-dissected antennae from Ringer solution into OCT Mounting medium for cryotomy (VWR, Radnor, PA, USA) and frozen at − 70 °C. Cryotomy was performed by plating 10 μm cryosections of dissected antennae deposited onto adherent glass slides using a Microm HM 560 Cryostat (Thermo Fischer Scientific, Waltham, MA, USA). Sections were immediately fixed in 4% PFA in PBS for 10 min and washed gently in PBS, twice for 10 min. For permeabilization and blocking, sections were permeabilized in PT solution for 30 min, and then transferred to a humidified chamber for blocking with PTS solution for 30 min. Next, 100 μl of primary antibody solution in PTS solution was pipetted onto each slide containing cryosectioned antennae, and incubated overnight at 4 °C. Subsequently, slides were washed three time for 10 min using PT solution on a shaker and blocked in PTS solution for 30 min. Slides were then incubated with secondary antibodies for 2 h at RT in the dark. Slides were washed three times for 5 min using PT solution and finally mounted with the use of 60 μl Vectashield under a coverslip. All covered slides containing antennal sections were stored at 4 °C prior to imaging.

Innexin immunostaining micrographs were captured using a cLSM 880 laser-scanning confocal microscope (Carl Zeiss, Oberkochen, Germany) using 10 × –63 × water immersion objectives (C-Apochromat, NA: 1.2, Carl Zeiss). Both whole antennal and sectioned antennal samples were captured in their entire depth via Z-stack optical sectioning using ZEN software (Carl Zeiss), which was also used to adjust fluorescence channel intensities (channel gamma and min–max intensities), produce maximum intensity projections, draw scale bars, and export colorblind-accessible raster images with individual and merged fluorescence channels.

### SSR electrophysiology

Single sensillum recordings (SSR) were performed on *D. melanogaster* and *D. suzukii* antennae prior to backfilling with tracer. Flies 2 + days old were held immobile in 200 μl pipette tips and fixed onto a glass side with laboratory wax. The third antennal segment was fixed in such position that the medial-posterior side faced the observer. Extracellular recordings were done using electrochemically (3 M KOH) sharpened tungsten electrodes by inserting ground electrode into the eye and recording electrode into the base of sensilla using the SM-10 micromanipulator system (Luigs & Neumann, Ratingen, Germany). Sensilla were then visualized with 1000 × magnification using an Olympus BX51WI binocular microscope (Olympus, Waltham, MA, USA). Signals were amplified using a Syntech Universal AC/DC Probe (Syntech, Buchenbach, Germany), sampled (96,000/s), and filtered (3 kHz high-300 Hz low, 50/60 Hz suppression) using a USB-IDAC. Neuronal activity was recorded using AutoSpike software (v3.7). The methyl acetate stimulus was delivered for 500 ms and was added to pre-humidified air in constant delivery onto the fly at a rate of 0.6 LPM. Methyl acetate (CAS: 79–20-9) was purchased from Sigma-Aldrich available at the highest purity. 10 μl methyl acetate was dissolved in hexane (1:10,000) and pipetted onto a filter paper of diameter 10 mm. Various large basiconic sensilla were recorded from to identify the antennal basiconic sensillum 2 (ab2) by methyl acetate response as best ligand/diagnostic odor for the sensillum in both species.

### Whole antennal backfills

Whole antennal backfills were performed by counterstaining antennae which were first excised whole into Ringer solution supplemented with 0.1% Triton X-100. Antennae were then stained by placement into excess 1–2% neurobiotin solution for 30 min at RT. Antennae were centrifugated using tabletop centrifuges and excess solution was siphoned off. Antennae were then washed with Ringer solution, immediately fixed in 4% PFA in Ringer solution for 30 min at RT, washed four times in PBS supplemented with 0.1% Triton X-100, and finally mounted in Vectashield for confocal imaging.

### Single sensillum backfilling

Following a positive identification of an ab2 sensillum by SSR, the same sensillum was backfilled by injection of NEUROBIOTIN Tracer (SP-1120, Vector Laboratories), a 323 Da tracer (more technically 286 Da upon dissolving due to loss of hydrochloride (Huang et al. 1992)) into the apical portion of the sensillum shaft by glass capillary. The SSR tungsten recording electrode was replaced with a pulled glass capillary which contained a replacement recording filament, where the capillary was pre-filled by capillary action with neurobiotin via dipping the unsharpened end of the capillary into a 2% (m/v) solution of the dye in 0.25 M KCl. Upon making a good recording contact wherein odor responses were evident, the contact was maintained for 45–90 min under periodic odor stimulation (every 5–10 min) with methyl acetate to help push the dye toward the antennal lobe. The procedure was performed on many flies and antennae, all of which would be saved for subsequent immunohistochemical treatment, and discarded when injected incorrectly.

Shortly following backfilling, antennae were excised from flies into *Drosophila* Ringer solution and immediately fixed for 2 h in 4% PFA in PBS on a shaker in 1.5-ml Eppendorf tubes (Eppendorf, Hamburg, Germany). Thereafter, antennae were washed four times for 15 min each in PBS supplemented with 0.1% Triton X-100, and incubated with 1:1000 streptavidin-Alexa Fluor 555 conjugate (S21381, Invitrogen, Thermo Fisher Scientific) fluorophore at 1:1000 dilution in PBS overnight at 4 °C. The next day, antennae were washed four times for 20 min and then mounted between microscope slide and coverslip in Vectashield for imaging.

Micrographs were captured for correctly backfilled antennae using a cLSM 880 confocal microscope using 10 × –63 × water immersion objectives (C-Apochromat, NA: 1.2, Carl Zeiss). Close-up images were obtained using the super-resolution Airyscan mode whereby scanning was performed using the Airyscan detector on the cLSM 880 (Huff 2015). Z-stack maximum intensity projection images were obtained from imaging planes (55 slices) at intervals of 200 nm over a 10.8-μm depth where backfilling immunofluorescence was observed. All confocal images were adjusted for contrast and brightness with ZEN software (Carl Zeiss). For 3D reconstructions, backfilled sensilla imaged using the cLSM 880 were reconstructed in 3D using the software Amira (Thermo Fischer Scientific).

## Results

### Innexins are abundantly expressed in drosophilid antennae

**Fig. 1:**
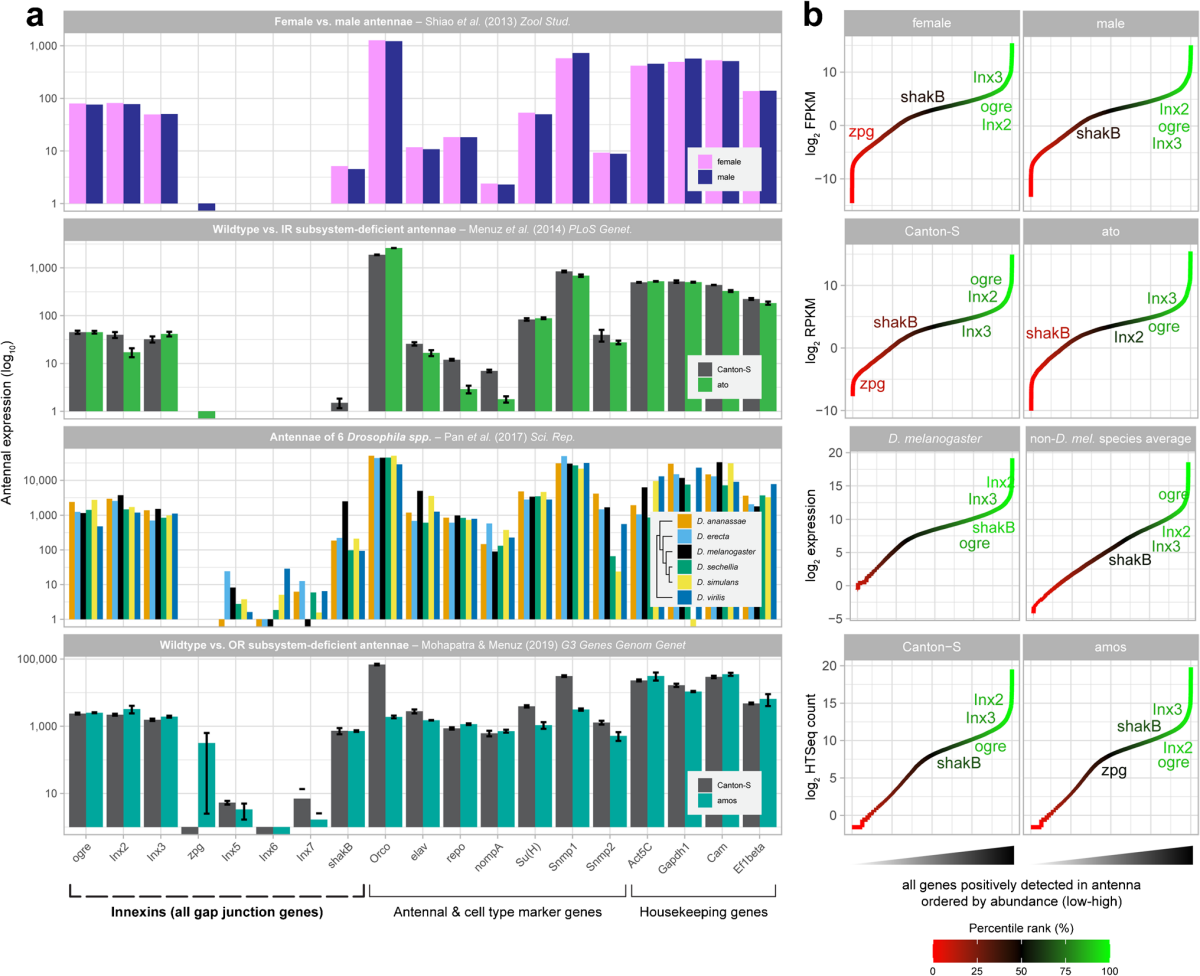
Innexin genes are abundantly expressed in *Drosophila* antennae. **a** Antennal transcriptomic expression profiles across four antenna-specific RNA-seq studies comparing expression of all *Drosophila* innexins to antennal genes of interest such as antennal cell markers and housekeeping genes. Studies include expression comparisons across sex, wildtype and mutant genotypes producing various olfactory subsystem deficiencies, and various *Drosophila* spp. for comprehensiveness. All studies exhibit transcriptomic profile similarity with regard to innexin expression. Where appropriate, error bars indicate standard error of the mean (SEM). **b** Corresponding scatterplots showing expression levels of all genes positively detected in antennal RNA-seq studies, ordered by expression abundance, and colored by their expression percentile rank among all genes detected expressed in the antenna. All detected innexin genes are labeled; gene labels are vertically displaced from where they appear. Genes not detected expressed in antennae are omitted

To begin determining whether gap junctions or hemichannels are present in the *Drosophila* antenna, we first asked whether innexin gene transcripts are expressed in antennal tissues of adult *Drosophila* flies. For this, we surveyed reference transcriptomic datasets from four RNA-seq studies sampling drosophilid antennae from various sources. For robustness, we consulted antennal transcriptomes comparing gene expression across fly sex, wildtype animals and olfactory subsystem mutants, and various *Drosophila* species to determine how variable or typical antennal innexin expression is relative to known antennal genes, cell type markers, and ubiquitous housekeeping genes. We considered all eight known *Drosophila* innexins in our search (Phelan et al. 1998a, b; Skerrett and Williams 2017), and plotted their expression for each study along with a common set of genes of interest, for reference. In order of appearance, antennal transcriptome studies included here feature comparisons between adult antennae sourced from male and female flies (Shiao et al. 2013); antennae from Canton-S wildtype flies and *atonal* (*ato*) mutants which lack coeloconic sensilla constituting the ionotropic receptor (IR) olfactory subsystem (Menuz et al. 2014); antennae from variously related drosophilid species *D. ananassae*, *D. erecta*, *D. melanogaster*, *D. sechellia*, *D. simulans*, and the distantly outlying species *D. virilis* (Pan et al. 2017); and antennae from Canton-S wildtype flies and *absent MD neurons and olfactory sensilla* (*amos*) mutants which lack basiconic and trichoid sensilla constituting the odorant receptor (OR) olfactory subsystem (Mohapatra and Menuz 2019). We found that the antennal presence of innexin transcripts was independently evident in all four studies, to degrees comparable with other well-studied olfactory and antennal genes, which were plotted for comparison, where *ogre*, *Inx2*, and *Inx3* consistently appeared abundantly, and where *shakB* also appeared substantially in most transcriptomes (Fig. 1a). *zpg* transcripts were also detected in the majority of studies, but were only detectable in some transcriptomes. Remaining innexins *Inx5*, *Inx6*, and *Inx7* were not detected in most antennal datasets (Fig. 1a).

In general, abundant innexins showed no differential expression across sex, genotype and species, nor variable expression within sample replicates, which may hint at a rudimentary and maintained function of innexins within antennae of adult flies. We found a particular exception to exist: *shakB* alone has been identified depleted (approx. fivefold) in *ato* mutant relative to wildtype antennae (Menuz et al. 2014), hinting that this particular innexin is more prominently expressed in coeloconic sensilla, or features thereof. On this front, we additionally consulted a more recent study directly comparing transcript expression across *ato* and *amos* mutant fly antennae, wherein either IR (coeloconic sensilla) or OR olfactory subsystem (composed of basiconic/trichoid sensilla) is known to be deficient, respectively, and found no significant depletion or enrichment among any of the eight *Drosophila* innexins genes above a fourfold-expression differential (Scalzotto et al. 2022). This corroboration suggests that innexin expression is not substantially restricted to either olfactory subsystem.

To gauge the degree of expression of innexins, we plotted the expression of all genes separately for each transcriptome available, ordered from low to high by expression magnitude (Fig. 1b). This was performed to determine more quantitatively whether the apparent abundances of innexins are substantive relative to all other positively expressed genes in the antenna. Here, *Drosophila* genes not expressed in the antenna were excluded for comparative insight. We found that *ogre*, *Inx2*, *Inx3*, and *shakB* transcripts were typically more numerous than genes of average antennal expression, and that *ogre*, *Inx2*, and *Inx3* were consistently abundant enough to occur within the top quartile of expressed genes. In almost all transcriptomes, *shakB* expression was found to occur within the interquartile range (considered medium abundance). Where detectable, *zpg* was found most frequently in the bottom quartile of genes by expression, and was presumed to be an antennally expressed innexin of low abundance. Notably, we also found no gross differences in innexin expression abundance across six surveyed *Drosophila* species and also in comparing innexin expression magnitudes between the model organism *D. melanogaster* with non-model drosophilid species (Fig. 1b).

We conclude that innexins are present in drosophilid antennae, and that their expression levels are considerable. Further, we specifically identify innexins *ogre*, *Inx2*, *Inx3*, and *shakB* as highly abundant antennal gene transcripts, and describe innexins *Inx5*, *Inx6*, and *Inx7* gene transcripts as absent from adult antennae. To a lesser extent, *zpg* was found detected in antennal tissues but did not appear in all transcriptomes surveyed, thus implicating it as a possible antenna-active gene. In sum, five of eight *Drosophila* innexins are implicated by transcriptomic expression as actively expressed within mature antennae of adult flies, with other remaining innexins considered non-antennal by exclusion.

### Antennal innexins are differentially expressed among cells and cell types of the antenna

**Fig. 2:**
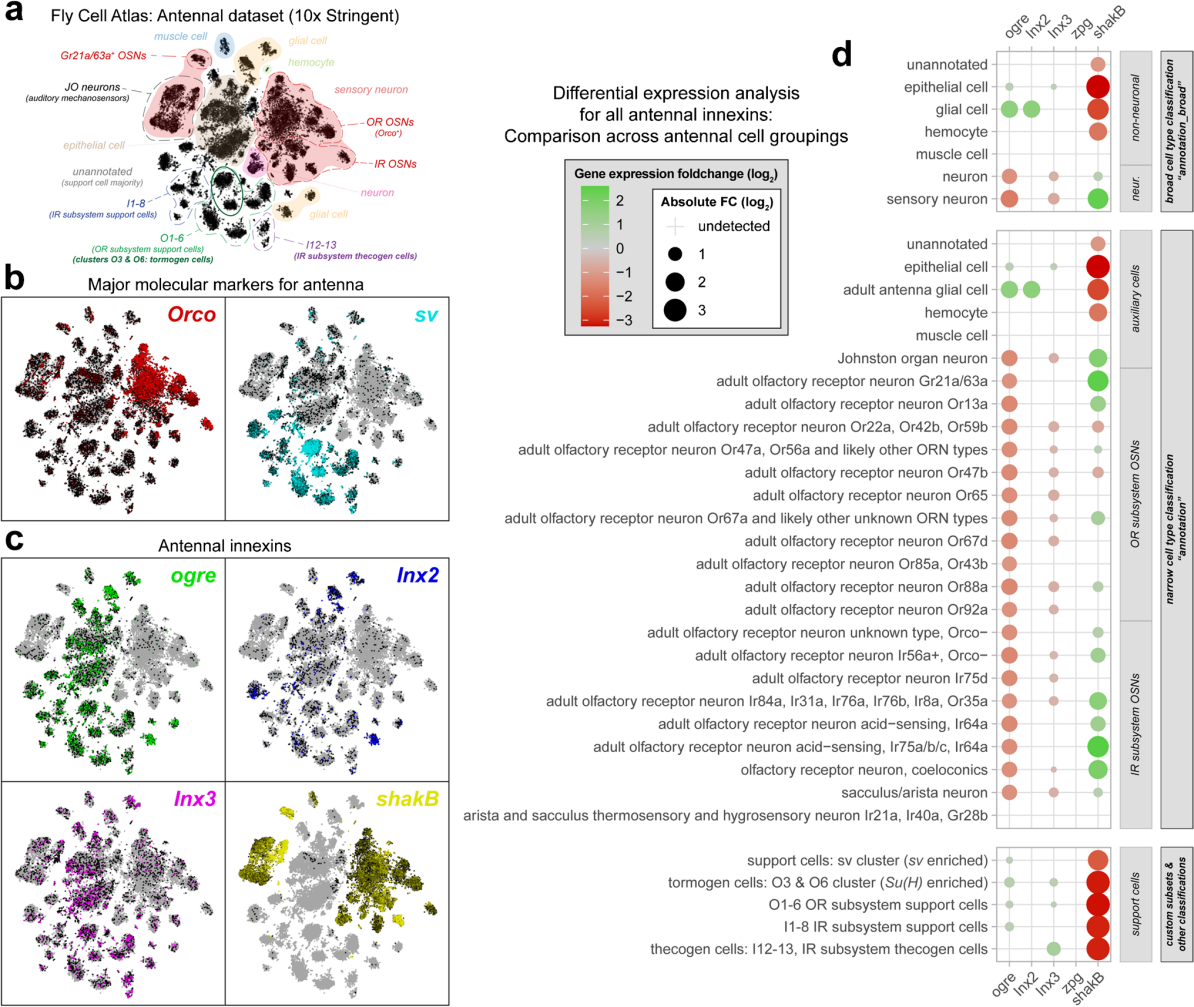
Cell type-specific expression of innexins as revealed by antennal tissue single cell RNA-seq. **a** tSNE coordinate visual representation of the Fly Cell Atlas’ antennal dataset (10X, stringent dataset) manually annotated with broad cell type (highlights) and known clusters (dashed outlines) based on various dataset annotations and literature. **b** Gene expression by color for two representative and major cell type-specifying markers. Left: OR subsystem OSN marker odorant receptor co-receptor (*Orco*); right: support cell marker shaven (*sv*). **c** Gene expression by color for all innexins reliably detectable in the antennal dataset. **d** Differential expression analysis of antennal innexins (*ogre*, *Inx2*, *Inx3*, the largely antennally absent *zpg*, and *shakB*) across three cell grouping schemes: broad cell type classification (top), narrow cell type classification (middle), and custom classifications based on existing literature (bottom). All differential expression comparisons consist of Wilcoxon rank-sum tests for specified cell groupings compared to the complementary set (i.e., all other antennal cells not within the tested group). Only statistically significant entries are plotted, colored by gene expression foldchange (differentially upregulated tending green, differentially downregulated tending red; magnitude of detected differential expression is represented by dot size). FC, foldchange

To further explore features of antennal innexin expression, we consulted single-cell transcriptomes of the *Drosophila* antenna. Specifically, we queried various antenna-specific datasets of the Fly Cell Atlas (McLaughlin et al. 2021; Li et al. 2022a), a resource that samples both neuronal and non-neuronal cells of the antenna with cellular resolution advantages unattributable to antennal datasets originating from bulk tissue RNA sequencing methodologies or single-cell approaches which include pre-filtering of cell types using fluorescence-activated cell sorting (FACS) (e.g., Li et al. 2020). The single-cell antennal dataset resolves major cell type groupings evident in *t*-distributed stochastic neighbor embedding (tSNE) visual representations which have been thoroughly but incompletely annotated (Fig. 2a). On a per-cell basis, the antennal dataset evidently contains both *Orco*^+^ OSNs and support cells, respectively expressing the corresponding cell markers *odorant receptor co-receptor* (*Orco)* and *shaven* (*sv*) (Fig. 2b). Here, we asked whether the distribution of innexin gene expression is cluster- or cell type-specific among all antennal cells. We found that all major antennal innexins exhibit highly heterogeneous expression (Fig. 2c). Namely, *ogre* expression appeared broadly non-neuronal and was absent from neuronal cell clusters such as IR- and OR-expressing OSNs, gustatory receptor (GR)-expressing OSNs such as *Gr21a/63a*^+^ OSNs, and Johnston organ (JO) mechanosensory neurons. *Inx2* expression was found limited principally to glial cell clusters, and in lesser extent to unannotated, epithelial and support cell clusters. Similarly, *Inx3* expression was largely non-neuronal with dispersed expression and greater abundance in epithelial cell clusters than *Inx2*. Lastly, *shakB* expression was strongly restricted to sensory neuronal cell subpopulations, most obviously in JO mechanosensors and IR subsystem OSNs, and proportionally lesser in OSNs of the OR subsystem which are *Orco*^+^ (Fig. 2c). Generally, the apparent degree of restriction or segregation of expression of some innexins to cell type groupings was striking to us, given that typical queries of gene expression among this dataset produce highly scattered expression patterning; this may be attributable to the abundance of innexin expression within the antenna, which produces sufficient patterning given available sequencing depth (Li et al. 2020, 2022a; McLaughlin et al. 2021).

To validate these impressions, we performed an extensive differential expression analysis for antennal innexins across several community cell grouping annotations. The analysis was performed on a per-innexin basis, considering broad (general) cell type classifications, narrow (finer) cell type classifications, as well as custom and recently proposed classifications of support cell clusters which have been partitioned across olfactory subsystems (Scalzotto et al. 2022). For differential expression testing, we selected to employ a false positive–conservative test, the non-parametric, two-tailed Wilcoxon rank-sum test (Li et al. 2022b), where the tests compare expression in each class with all other cells of the dataset (i.e., complementary set). The results for all statistically significant differential expressions are summarized in a differential expression analysis table showing foldchange in expression between cell groups (Fig. 2d). In short, *ogre* was found significantly enriched in epithelial, glial, and support cells, and depleted in (sensory) neurons, a finding evident across all more fine-grained OSN categorizations. In turn, *Inx2* was found enriched solely in glial cells. *Inx3* was found enriched in epithelial and support cells, and depleted in all neuron classes. Unlike *ogre*, *Inx3* was enriched in thecogen cell subpopulations and showed no enrichment within the glial cells grouping. Finally, and in line with expectations, *shakB* was found enriched in (sensory) neurons alone, and was correspondingly and strongly depleted elsewhere, including the ill-defined, large and heterogeneous “unannotated” cell class (Fig. 2d).

Intriguingly, there exists considerable variability in *shakB* expression among OSN subclasses: *shakB* was depleted in groups consisting of large olfactory neurons of large basiconic sensilla—those expressing *Or22a* (*odorant receptor 22a* serving as a unique marker for the neuron of the antennal basiconic sensillum 3, i.e., ab3A), *Or42b* (ab1A neuron), and *Or59b* (ab2A neuron)—as well as in the large neuron of a trichoid sensillum expressing *Or47b* (at4A neuron). For most other olfactory neuron groupings, *shakB* was relatively enriched, irrespective of olfactory subsystem. We therefore note the heterogeneity of *shakB*-specific innexin expression among OSNs specific to the olfactory periphery of *Drosophila*.

For robustness, sister datasets originating from variant data pre-processing, annotation schemes, and single-cell capture methods prior to sequencing have also been analyzed in tandem for comparison. Here, comparable differential expression patterns were evident, and these results were found to hold similarly across all antennal innexin genes (Supplementary Fig. S1). Lastly, in agreement with bulk antennal tissue transcriptomic data presented prior, *Inx5*, *Inx6*, and *Inx7* remain entirely absent from all single-cell antennal datasets, while *zpg* was detected in trace quantities uninformative for analysis.

### Whole antennal immunofluorescence stainings reveal differences in innexin labeling

To supplement bioinformatic insights into the expression of innexin genes in the *Drosophila* antenna, we next opted to investigate potential gap junction or hemichannel localization by use of immunochemical stainings using innexin-specific antibodies to characterize antennal innexins on the protein level. Here, we employed previously validated antibodies specific to the previously-shortlisted antennal innexin proteins ogre, Inx2, Inx3, and shakB that have been used in *Drosophila* (Phelan et al. 1998b, 2008; Bauer et al. 2003, 2004; Bohrmann and Zimmermann 2008; Holcroft et al. 2013; Smendziuk et al. 2015; Richard et al. 2017; Ammer et al. 2022). To this end, we first collected intact antennae from live flies expressing membrane-targeted (mCD8) green fluorescent protein (GFP) in Orco^+^ OSNs by way of Gal4/UAS binary expression (Lee and Luo 1999). Multiple antennae were immunostained following antennal dissection and imaged in their entirety using a two-channel confocal fluorescence microscopy to capture GFP- and innexin antibody–associated fluorescence separately. Subsequently, all specimens, optical sections as well as maximum intensity projections were carefully examined.

**Fig. 3:**
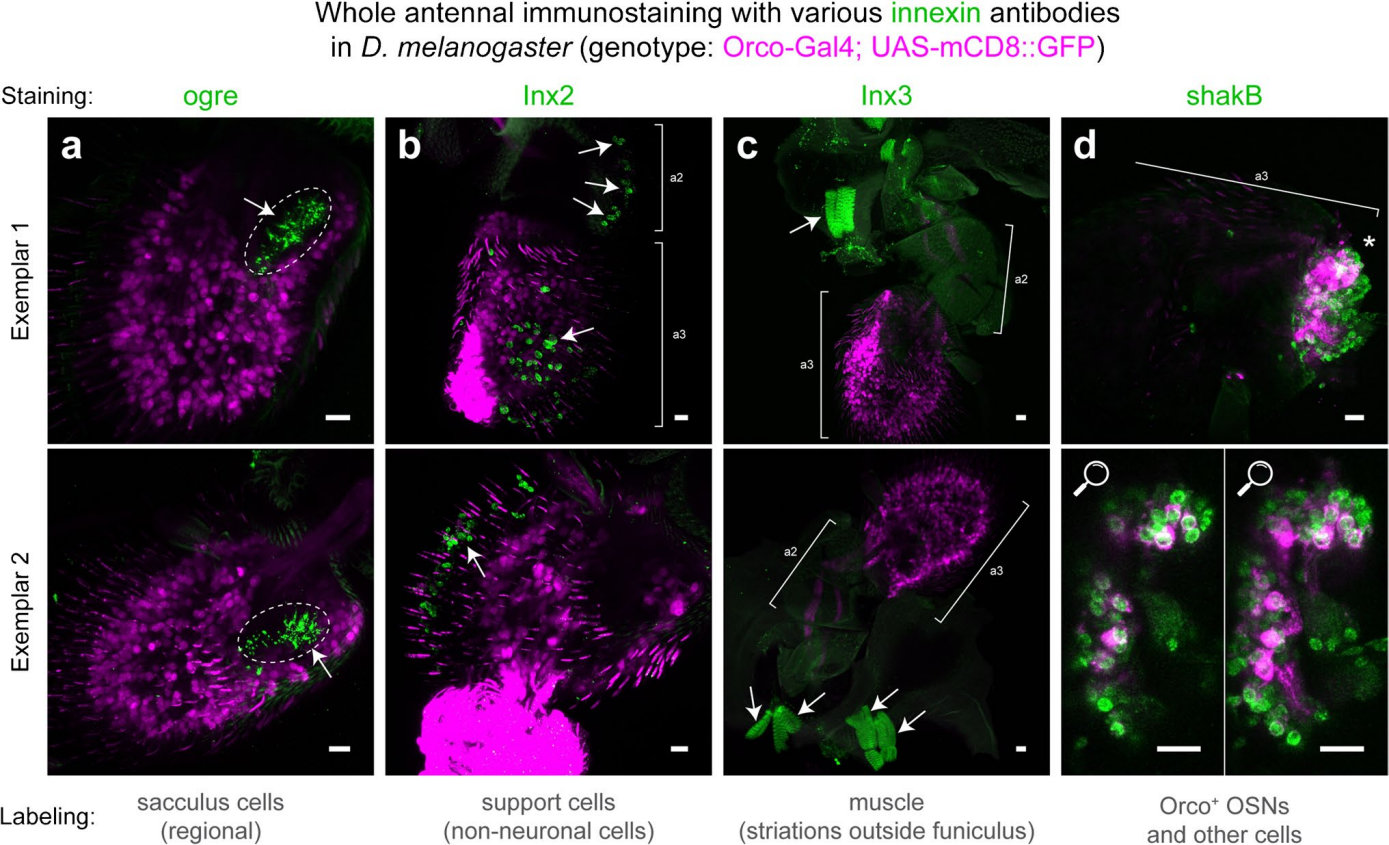
Immunofluorescence staining of innexins in whole antennal samples. Intact, dissected antennae from *D. melanogaster* expressing GFP in Orco^+^ OSNs via Gal4/UAS binary expression system, were stained separately with innexin antibodies. Two exemplary micrographs are provided for each immunostaining, namely of ogre (**a**), Inx2 (**b**), Inx3 (**c**) and shakB (**d**). Apparent localization is summarized (bottom). Arrows indicate stained cellular structures. Dashed lines outline the sacculus region. a2: second antennal segment (pedicel). a3: third antennal segment (funiculus). A single asterisk (*) indicates region where the funiculus stem is detached from the pedicel, for which magnifications at different optical planes are provided below. All scale bars: 10 µm

Upon imaging, we discovered unique immunostaining patterns for each of the four antennal innexins, over several immunostaining replicates pooling antennae from 30 to 100 flies. In samples immunostaining ogre, we repeatedly observed strong staining around the sacculus pits found in the funiculus (Fig. 3a), which houses coeloconic sensilla of the olfactory IR subsystem responsible for hygroreception, thermoreception, and chemoreception of acids, free protons, ammonia, and (poly)amines (Shanbhag et al. 1995; Silbering et al. 2011; Enjin et al. 2016; Prieto-Godino et al. 2017; Rimal and Lee 2018).

In contrast, antennal immunostaining of Inx2 showed staining of globular cell-like structures in the pedicel and funiculus, which did not co-localize with any signal from the Orco^+^ OSN fluorescence channel (Fig. 3b). Localization in both antennal segments and the apparent morphology and distributed positioning suggests that these labeled cells or structures are non-neuronal but rather auxiliary, such as epithelial or support cells, which are of more rounded shape and known to occur in similar fashion in both antennal segments due to cellular homology between chordotonal organs and olfactory sensilla (Shanbhag et al. 2000; Chung et al. 2001; Prelic et al. 2022). Interestingly, the localization was not widely uniform across the funiculus but rather distally distributed and ipsilateral to the arista (Fig. 3b). Based on antennal location, we guessed these cells may be located in trichoid or intermediate sensilla characteristic (though not exclusive) of this area (Shanbhag et al. 1999; Lin and Potter 2015). Hence, we repeated Inx2 stainings using antennae from flies expressing GFP in antennal trichoid sensilla, in *at1* and *at4* trichoid neurons, using *Or67d-Gal4* and *Or47b-Gal4* driver lines respectively, but found no obvious evidence of colocalization with these neurons and the corresponding trichoid sensillum type (Supplementary Fig. S2).

Next, we observed that Inx3 immunostaining did not produce any discernable signals in the antennal pedicel or funiculus segments, in a consistent manner across all imaged antennae. However, where co-sampled, Inx3 immunolabeling showed surprisingly strongly stained striated structures beyond these antennal segments (Fig. 3c). We presume these striated structures to be instances of labeling of gap junctions integral to insect and invertebrate muscle tissue (Liu et al. 2006, 2013; Yoshimura et al. 2017; Güiza et al. 2018) that were co-sampled along with these segments during antennal excision from the *Drosophila* head in sample preparation. Regardless of the exact nature of the striations, we do not consider these structures to be antennal and leave the micrography open to interpretation.

Finally, whole antennal immunostainings for shakB showed patchy overlap with the GFP fluorescence channel labeling Orco^+^ OSNs (Fig. 3d). In spite of this obvious co-localization with Orco^+^ OSNs, we were unable to clearly discern whether shakB signal was also localizing to or absent from any non-neuronal cells in these preparations. Apparent presence of shakB in a subset of OSNs at the protein level is in line with expectations based on our prior transcriptomic evidence of patchy expression of *shakB* transcripts in some, but not all OSN classes.

### Innexin immunostaining in sectioned antennae reveals specific localization of innexins at cellular resolution

Contrary to expectation, we noticed most attempts of immunostaining of samples of whole, intact antennae did not mark internal structures within the antennal segments in detail, an observation that is in conflict with estimations of localization or gene expression from our prior antennal transcriptomic inferences. For instance, due to the strong enrichment of *ogre* transcripts in glial cell subpopulations, we expected ogre immunostainings to overtly mark or in part localize to glial sheaths of axonal projections, and render the known anatomical structure of antennal glia (Sen et al. 2005; Prelic et al. 2022; Calvin-Cejudo et al. 2023). In most instances, we speculated that despite the long duration of preparatory permeabilizing and incubation with antibodies during sample preparation, the perfusion of the antibodies into the antennal tissue was restricted, partial or in some cases seemingly non-existent. We base this presumption threefold. First, the cuticle is a known and major penetration barrier to chemical or even pharmacological treatments such as protease digestion or pharmacological activation/inhibition of physiological properties of neurons, and as such usually requires antennal experiments like functional imaging investigations to expose antennal tissue by opening the antenna (Cao et al. 2016; Halty-deLeon et al. 2018; Jain et al. 2021; Miazzi et al. 2022; Prelic et al. 2023). Due to the relatively large size of antibodies over small enzymes or pharmacological agents, it is likely that immunochemical penetration may have been mitigated and produced incomplete stainings in intact antennal preparations. Second, we observed that antennae with nicked cuticles were more successfully stained, or that cells adjacent to entrances into the funiculus were stained (e.g., Fig. 3d). Thirdly, we have previously reported incomplete permeation of antibodies into intact antennal samples observed via immunofluorescence (Prelic et al. 2022). Whole antennal immunostainings were therefore presumed to mark readily accessible parts of the antenna, which may have been rendered open to immunostaining through nicking or damaging of the cuticle during sample preparation.

To circumvent this limitation in tissue accessibility to antibody perfusion, we next opted to prepare thin sections of antennal tissue using cryotomy sectioning. Cryosectioned antennae underwent a similar immunostaining procedure as before. In parallel with this approach, we were additionally interested in whether innexins may localize to specific support cells, a heterogeneous and poorly understood set of cells which make up a significant fraction of cells in the antennal appendage (Shanbhag et al. 1999, 2000; Prelic et al. 2022). Thus, we also further employed independent labelings of support cell subtypes via previously described support cell markers to aid in better discerning the apparent localization of antennal innexins. For this, we employed *nompA-Gal4* and *ASE5-Gal4* fly lines to drive GFP reporter expression separately in thecogen and tormogen cells, respectively (Barolo et al. 2000; Chung et al. 2001; Prelic et al. 2022).

**Fig. 4:**
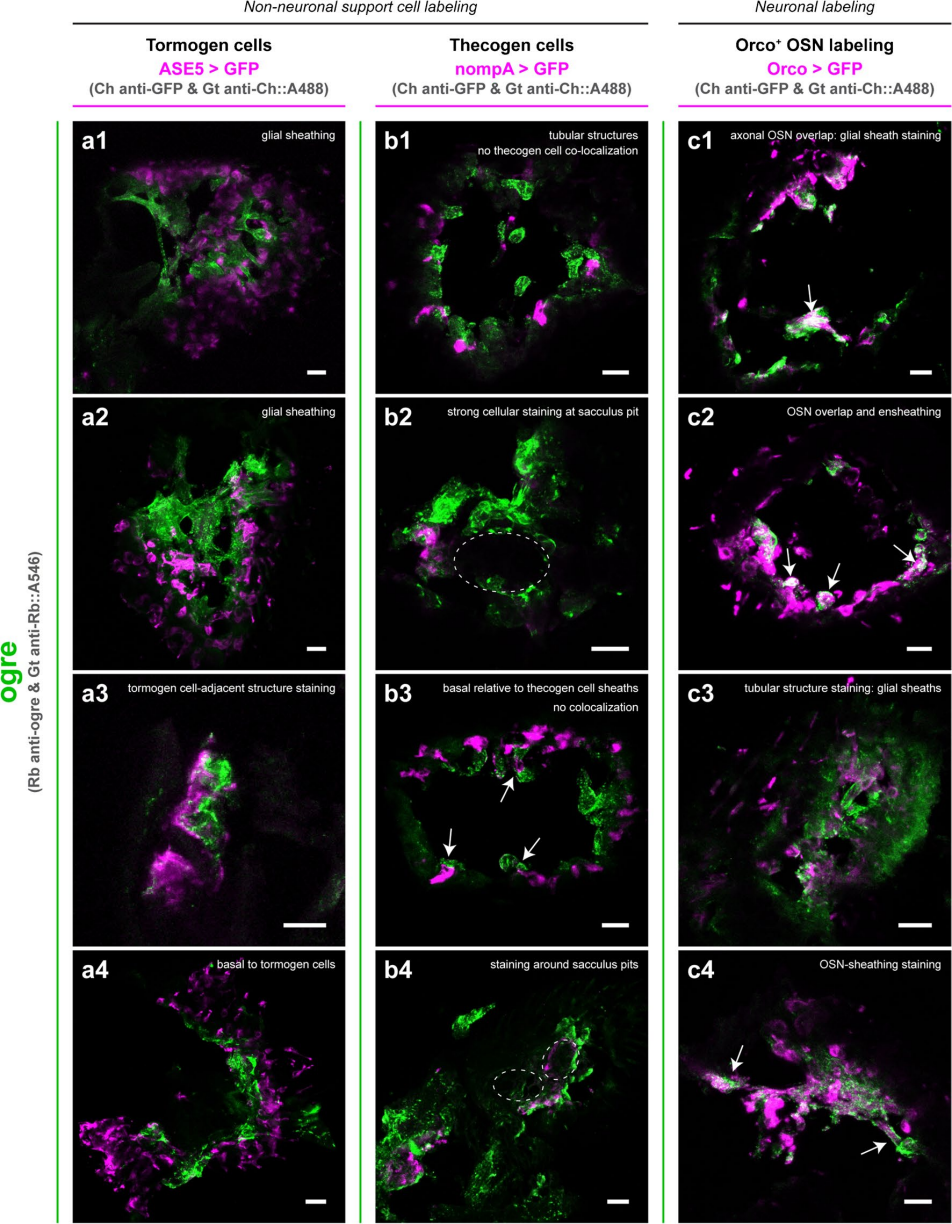
Ogre immunofluorescence staining in cryosectioned antennal tissue with support cell and OSN counterstaining. As a colocalization study, ogre immunostaining was performed in sections of antennae expressing Gal4-driven fluorescent labeling of tormogen socket cells (**a**), thecogen sheath cells (**b**), and Orco^+^ OSNs (**c**). For each counterstaining (**a**, **b**, **c**), four exemplars (1–4) are provided. Observations of ogre protein localization are summarized for each panel (arrows point to relevant structures described within the panel). Dashed lines outline sacculus pit regions. Ch, chicken; Rb, rabbit; Gt, goat. All scale bars: 10 µm

In antennal sections stained for ogre, we were able to observe denser and more complex staining (Fig. 4). Altogether, ogre staining marked glial sheath-like structures, and did not colocalize with marked tormogen (Fig. 4a) and thecogen (Fig. 4b) support cells, nor with somata of Orco^+^ OSNs (Fig. 4c). Localization of ogre was observed to closely follow OSN axons in a manner indicative of filamentous/tubular ensheathing structures of antennal glia (Sen et al. 2005), and was also basal relative to the more apical sensillum shafts, where most glial cell bodies reside. No evidence of colocalization with support cells was evident in any of the numerous sections imaged, though ogre-labeled structures appeared to be adjacent and sometimes confluent with both tormogen and thecogen cell labelings. No overlap between somata of Orco^+^ OSNs and ogre was evident, though partial overlap with OSN labeling can be seen, likely indicative of ogre localization to glial sheaths of OSN axons. We were also able to recapitulate prior ogre staining results of intact antennae, finding strong staining of structures around the sacculus pit, which do not feature Orco^+^ OSNs (Fig. 4b).

**Fig. 5:**
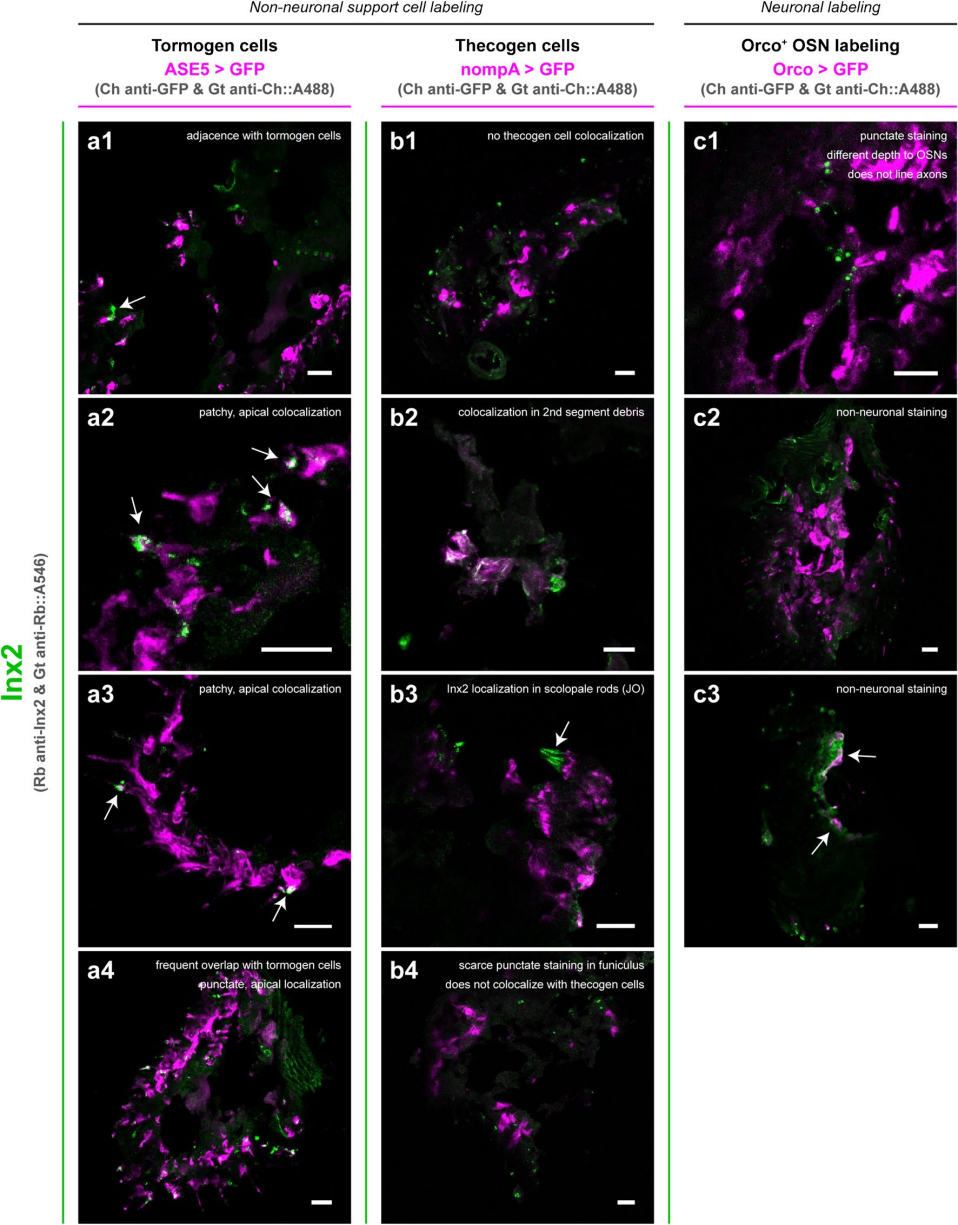
Inx2 immunofluorescence staining in cryosectioned antennal tissue with support cell and OSN counterstaining. As a colocalization study, Inx2 immunostaining was performed in sections of antennae expressing Gal4-driven fluorescent labeling of tormogen socket cells (**a**), thecogen sheath cells (**b**), and Orco^+^ OSNs (**c**). For each counterstaining (**a**, **b**, **c**), four exemplars (1–4) are provided. Observations of Inx2 protein localization are summarized for each panel (arrows point to relevant structures described within the panel). Ch, chicken; Rb, rabbit; Gt, goat. All scale bars: 10 µm

In antennal sections stained for Inx2, we observed much more punctate and scarce staining (Fig. 5). Inx2 signal often overlapped with tormogen cells and/or marked cell-like structures directly apposed to tormogen cells (Fig. 5a). Inx2 was not found to colocalize with thecogen cells in the funiculus of any examined antennal section, although appeared to closely stain together with *nompA-Gal4*-driven GFP in structures in the pedicel (Fig. 5b). In the pedicel, the thecogen cell-marking *nompA-Gal4* element is known to drive reporter expression in scolopale cells, homologous counterparts to the third antennal segment’s thecogen cells (Roy et al. 2013; Prelic et al. 2022). Scolopale cells are constituent cells of scolopidia, mechanosensitive units composing the Johnston’s organ (Chung et al. 2001; Roy et al. 2013; Boekhoff-Falk and Eberl 2014). Based on the thin, parallel-lined appearance, we interpret this Inx2 signal as indicative of this innexin’s localization within the scolopale rods, perhaps lining the neuronal dendritic membranes, or the cap or scolopale cell membranes. To our knowledge, innexin involvement local to scolopidia of the Johnston’s organ has not yet been described, and may be of interest for research concerning *Drosophila* audition and the Johnston’s organ. Inx2 did not appear to colocalize with the Orco^+^ OSN counterstain nor line OSN axonal projections (Fig. 5c).

**Fig. 6:**
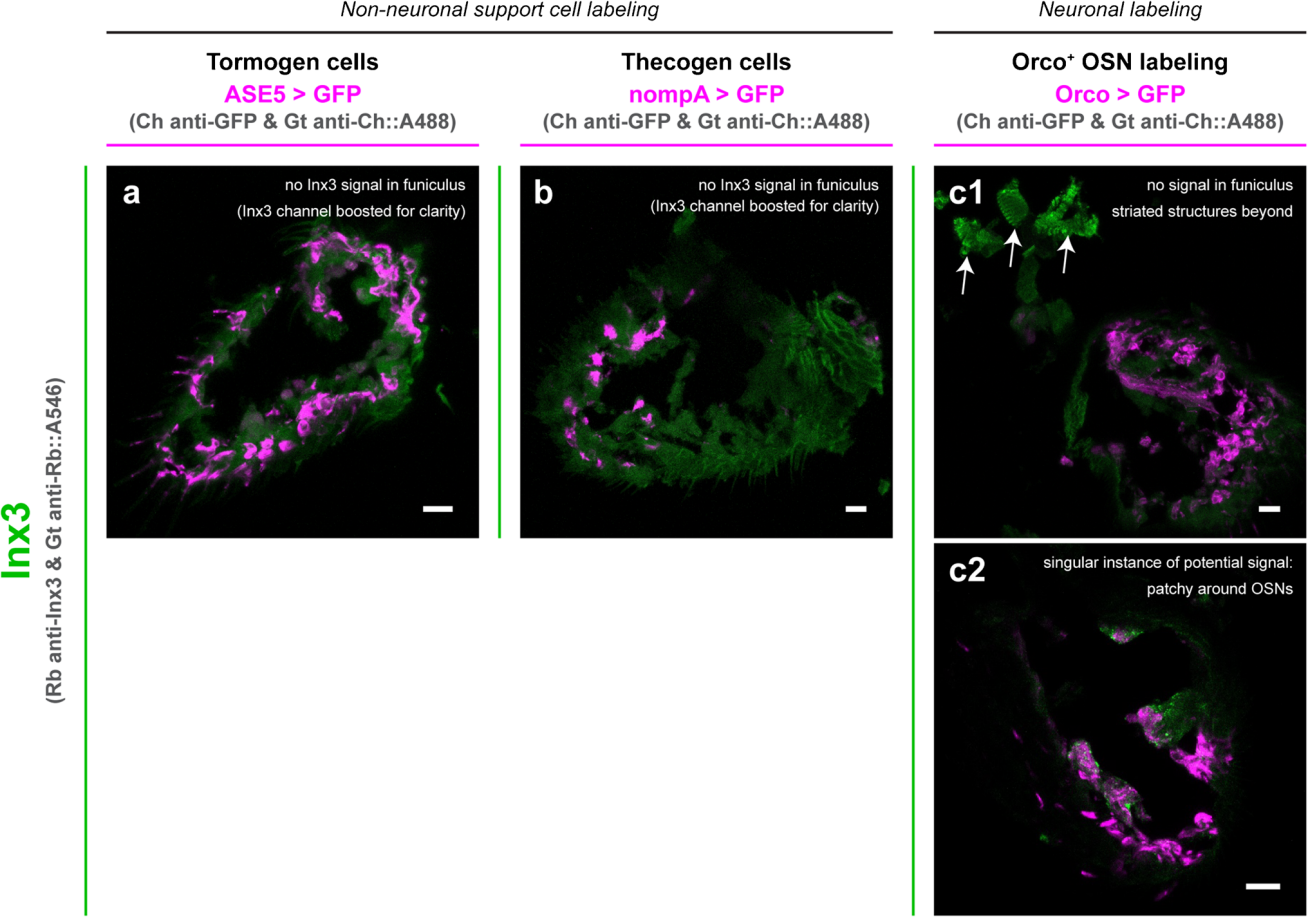
Inx3 immunofluorescence staining in cryosectioned antennal tissue with support cell and OSN counterstaining. As a colocalization study, Inx3 immunostaining was performed in sections of antennae expressing Gal4-driven fluorescent labeling of tormogen socket cells (**a**), thecogen sheath cells (**b**), and Orco^+^ OSNs (**c**). A representative exemplar is provided for each counterstaining. No staining is observed in the funiculus, though striated structures co-sampled along with second and third antennal segments are strongly Inx3-stained. Observations of Inx3 protein localization are summarized for each panel (arrows point to relevant structures described within the panel). Ch, chicken; Rb, rabbit; Gt, goat. All scale bars: 10 µm

Sections stained for Inx3 generally showed no trace of signal within the funiculus among almost all sectioned antennae examined (Fig. 6). Exceptionally, in the funiculus of one antennal section, we found a singular instance of punctate staining that did not overlap with the Orco^+^ OSN counterstain (Fig. 6c), in agreement with predictions based on non-neuronal *Inx3* expression inferred from single-cell transcriptomic analysis. However, we did not find a replicate of such a staining and thus consider the observation tentative. Last, we yet again found a striated Inx3 patterning in structures beyond the relevant (second and third) antennal segments (Fig. 6c) as observed in whole antennal mount preparations previously.

For completeness, we finally also employed immunostaining of zpg in antennal sections, as well as negative immunostaining controls featuring mismatched antibodies to gauge degree of background staining (noise). For both conditions, we hypothesized these would likely yield entirely negative staining results; in the case of zpg, with expectations of trace amounts of protein based on lack of detectable *zpg* expression in many antennal transcriptomes surveyed, and in the case of the negative control, expectations of unspecific background staining levels. Surprisingly, we found rich cellular staining for zpg (Supplementary Fig. S3). In short, zpg was found to label large, globular and mutually confluent cells closely apposed to tormogen cells. zpg labeling did not colocalize with any cell marker counterstain, including those of tormogen cells, and was not restricted to any portion of the funiculus, suggesting that zpg is specific to structurally large, common, and ubiquitously distributed cells, perhaps epithelial or trichogen cells. Lastly, fully in line with expectations, we found no positive signal for the negative immunostaining control experiment involving antibody mismatch (Supplementary Fig. S4). Images of these sections were used to determine typical noise levels of unspecific background staining, given the choice of secondary antibody common to all other stainings, and as such were used as reference controls for all other microscopic experiments involving immunofluorescence of antennal sections.

### Backfilling antennae and single sensilla using gap junction–permeable tracer dye

A previously suggested and intriguing possibility is that gap junctions may functionally interconnect support cells among themselves to aid in transcellular solute flow and facilitate the electrical properties of each sensillum unit (Thurm and Kuppers 1980; De Kramer et al. 1984; De Kramer 1985), interconnect neighboring neurons of multineuronal sensilla, and serve as a putative explanation of so-called “ephaptic inhibition” observable between neurons (Zhang et al. 2019; Pannunzi and Nowotny 2021), or mediate intercellular communication or coupling which may explain synchronized physiological activity between cells of the sensillum during odor detection (Prelic et al. 2022).

**Fig. 7:**
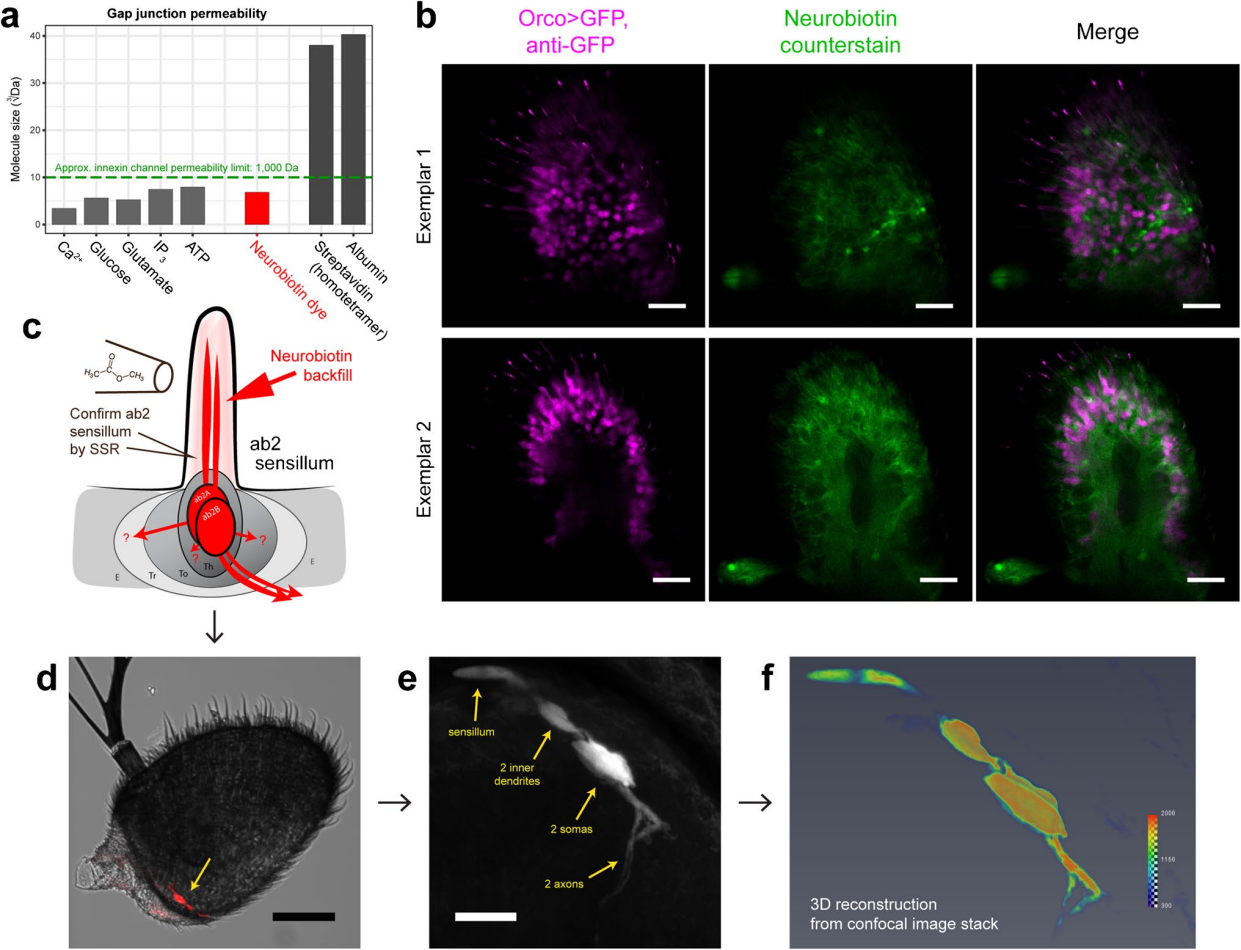
Antennal and single-sensillum backfilling using gap junction-permeable tracer dye. **a** Molecular size of compounds known to permeate via gap junctions (left bars), neurobiotin dye (middle, red), and compounds that cannot permeate gap junctions (right bars), expressed in cube-root of size in daltons to approximate three-dimensional size. Neurobiotin falls below the innexin channel pore size of 1 kDa, indicating dye permeability and suitability for gap junction tracing by dye coupling. **b** Third antennal segments exposed to uptake dye in a neurobiotin bath. Two exemplars are provided; all fluorescence imaging channels are shown. Tracer does not diffuse into neurons or neuronal compartments and remains segregated. All scale bars: 20 µm. **c** Schema of single sensillum backfilling experiment. Upon diagnostic identification of a specific two-neuron sensillum (ab2-like) using electrophysiological recording of diagnostic odorant responses (methyl acetate), a glass capillary is employed to deliver neurobiotin dye into the tip of the identified sensillum. Neuronal uptake and anterograde dye tracing is then promoted by periodic odor stimulation. The neurobiotin neuronal tracer is hypothesized to diffuse across cell boundary if existing gap junctional connections or hemichannels are present, and label other cells or compartments such as sensillum lymph. ab2A/B: large and small olfactory sensory neurons in ab2 sensillum. Th, thecogen cell; To, tormogen cell; Tr, trichogen support cell; E, epithelial cell. **d** Confocal micrograph of dissected funiculus showing tracer uptake by cells of a single sensillum at the proximomedial region of the funiculus (yellow arrow), opposite the arista. Scale bar: 50 µm. **e** Confocal maximum intensity projection at cellular resolution shows complete dye uptake by ab2A and ab2B neurons; all structures including sensory dendrites, soma and afferent axons are dyed with tracer (yellow labels). Scale bar: 10 µm. **f** 3D reconstruction of tracer’s fluorescence signal for high-resolution inspection. No tracer permeation or leakage across the neuron boundaries to any adjacent cells is evident

To functionally test whether cytoplasms or lymph of adjacent cells or sensillum compartments are made continuous due to interconnection via hemichannels, gap junctions or electrical synapsing, we designed an experiment employing a small-molecular weight dye capable of diffusing across insect gap junctions composed of innexin channels (Fan et al. 2005; Wörsdörfer and Willecke 2017). Small tracer techniques and “dye coupling” experiments exploiting the properties of permeability across junctions and impermeability across cell membranes have been classically employed for identifying gap junctions and cell–cell interconnections in a variety of organisms and tissues (Zimmerman and Rose 1985; Wörsdörfer and Willecke 2017). For our tracing purposes, we chose neurobiotin (Huang et al. 1992), a synthetic non-toxic 323 Da tracer derived from biotin that can be detected and subsequently visualized with streptavidin-conjugated fluorophores. Neurobiotin is of similar size to electrical synapse–permeable molecules such as inositol 1,4,5-trisphosphate (IP_3_) (Decrock et al. 2012) and ATP (Bao et al. 2007; Anselmi et al. 2008; Güiza et al. 2018), and is smaller than the 1–2-kDa gap junction size threshold in insects (Loewenstein 1981; Zimmerman and Rose 1985; Ammer et al. 2022) (Fig. 7a). Furthermore, it possesses advantages such as improved solubility and stability compared to similar tracers like biocytin and is thus well suited for use in sensitively tracing unknown, direct intracellular connections (Huang et al. 1992; Wörsdörfer and Willecke 2017). Among other systems, neurobiotin backfilling has been successfully employed in *Drosophila* tissues, including antennal auditory sensory neurons (Pézier et al. 2014, 2016; Jezzini et al. 2018; Ammer et al. 2022).

In our study, we opted to perform two opposite and complementary backfills using neurobiotin. In the first “control” approach, to rule out a possibility of unselective diffusion, we wanted to confirm that neurobiotin dye itself does not perfuse across the sensillum lymph/hemolymph compartment barrier. These compartments are thought to be strictly isolated from one another by support and epithelial cells, which tightly envelop OSNs and the sensory lymph with the aid of septate junctions, insect analogues of vertebrate tight junctions, thus allowing for tight control of water and solute flow (Noirot-Timothée et al. 1978; Steinbrecht 1980; Keil and Steinbrecht 1983, 1987; Shanbhag et al. 1999, 2000; Beyenbach 2012; Jonusaite et al. 2016; Nava Gonzales et al. 2021; Prelic et al. 2022). Here, we exposed intact third antennal segments to a neurobiotin solution bath for passive uptake, to trace the extent and path of dye diffusion through the antennal stem and hemolymph. Upon visualizing neurobiotin with a fluorophore conjugated to the highly (neuro)biotin-specific streptavidin, we observed broad and sufficient antennal perfusion and a characteristic dye patterning wherein sensillum compartments were left selectively unstained, as expected (Fig. 7b). These dye-free compartments were evenly distributed across the entire funiculus, and contained Orco^+^ OSNs, indicating that neurobiotin diffuses efficiently across the tissue but is not permeable across the sensillum lymph/hemolymph compartment divide. In our neurobiotin counterstaining, some cells were observed to have taken up neurobiotin more densely; these cells were not Orco^+^ OSNs (Fig. 7b). This result corroborates with older electron microscopic studies employing lanthanum ion tracers in lepidopteran sensilla, where perineuronal compartments were observed inaccessible to tracer diffusion (Keil and Steinbrecht 1987).

In our second backfilling approach, we aimed for more precise delivery of dye into a pre-determined, specific sensillum itself for neuronal uptake, as is typically performed for anterograde tracing of neuron projections, including those found within insect chemosensory sensilla (Anton et al. 2003; Ghaninia et al. 2007; Keesey et al. 2019; Saleeba et al. 2019). We hypothesized that precision-delivered neurobiotin into the sensillum lymph would be taken up by sensory neurons and diffuse throughout all continuous structures, tracing all latent intercellular connections, if present. With gap junctions present between neurons and tightly apposed support cells, the dye would diffuse visibly into neighboring contiguous cells. Alternatively, if hemichannels were present on neuronal membranes, connecting them to the sensillum lymph, we would expect to see dye diffusion tracing the well-isolated sensillum lymph compartment. For this purpose, we selected the typical, large, accessible, and well-studied multineuronal ab2 basiconic sensillum as our backfilling target. In preparation, we performed live single sensillum recording (SSR) in wildtype specimens of a few fly species to specifically identify the ab2 or ab2-like sensillum by probing individual sensilla for their electrophysiological response to air pulses of methyl acetate, the diagnostic odorant for the ab2 sensillum and best ligand of the Or59b receptor found in the ab2A neuron within this sensillum (Hallem and Carlson 2006; Guo et al. 2017; Keesey et al. 2019). Upon successful SSR-guided targeting, we then injected the same sensillum shaft with neurobiotin solution using a microcapillary (Fig. 7c). Contact with the sensillum shaft was maintained for 45–90 min under periodic odor stimulation with methyl acetate to help push dye uptake. For each attempt, antennae were imaged using confocal microscopy to screen for correct backfilling, i.e., where sensillum shafts were accurately punctured but not pierced through, where dye was restricted to a single sensillum, and where sufficient dye was applied. Fulfilling all criteria, we succeeded in correctly backfilling a *D. suzukii* ab2-like sensillum (Fig. 7d); the neurobiotin-backfilled animal was subsequently used in parallel for a neuronal anterograde tracing study screening OSN innervation in the antennal lobe, where neurobiotin diffusion was confirmed to have traced the entire distance of OSN axonal projections to the antennal lobe of the brain (Keesey et al. 2019). Here, we saw complete tracer confinement to the two neurons of the sensillum, and did not observe nor trace any dye diffusion across any neuronal membrane upon detailed inspection with high magnification confocal microscopy across the entire depth of the sensillum (Fig. 7e). We additionally performed a 3D reconstruction of the tracer signal for closer spatial inspection, and found no leakage past the sharply defined cell boundaries of the neuron pair (Fig. 7f). We therefore found no evidence of latent gap junction connections between neurons and support cells, nor hemichannels present on these OSNs. The observation does not exclude the possibility of interconnectedness between the two neurons of the ab2 sensillum. Due to difficulty of simultaneous success in diagnostic electrophysiological measurement and correct backfilling of the same sensillum (of approx. 1–2 µm diameter), we were unable to repeat a comparably successful backfilling in *D. melanogaster* for comparison.

## Discussion

This study presents a first insight into differential expression and localization of innexins in the *Drosophila* antenna. We report the active and maintained presence of innexins *ogre*, *Inx2*, *Inx3*, *zpg*, and *shakB* in chemosensory sensilla within the funiculus, the third antennal segment of the antennal appendage largely responsible for olfactory perception. A summary of observed localization across transcriptomic and immunohistochemical stainings of several innexins in intact and sectioned antennae is provided in Table 2. In a recent comprehensive mapping of innexins in the *Drosophila* brain, innexins *ogre*, *Inx2*, and *Inx3* were found to be exclusively glial (Ammer et al. 2022). We have found this pattern does not hold fully in the antenna, as in several cases innexin transcript enrichment was found in both non-glial and non-neuronal cells such as tormogen support cells. We therefore caution that gap junction and/or innexin localization cannot be assumed by extrapolation based on innexin studies in other neuron-dense tissues of *Drosophila*. We also report the expression of the innexin *shakB* in antennal OSNs. Given the descriptive and exploratory nature of the presented data, we further discuss important considerations and several lines of interpretations and suggested hypotheses which follow from our observations amid existing literature.

**Table 2:** Summary of transcript expression and protein localization determined by antennal scRNA-seq and immunostaining of all antennal innexins within the *Drosophila* antenna

Antennal innexin	scRNA-seq localization	Whole mount antennal localization	Localization in sectioned antennae
*ogre* (*Inx1*)	- Glial, epithelial, support cells, tormogen cell subset - Non-neuronal	- Sacculus region	- Likely glial localization, stains sheath structures - Localization excludes OSNs, tormogen, and thecogen cells - Basal localization (sensillum shaft-distant) - Stains cells closely apposing tormogen and thecogen cells (epithelial and trichogen cell localization likely) - Enrichment around sacculus region
*Inx2*	- Glial	- Subset of (globular) support cells - Distributed distally in funiculus	- Localization apical, closer to sensillum shafts - Colocalizes with tormogen and apical support cells - Localization excludes thecogen cells - Unlikely neuronal or glial - Marks scolopale rods (2nd segment, JO)
*Inx3*	- Glial, epithelial, support cells, tormogen cell subset - Non-neuronal	- Not in funiculus - Muscle/striated tissue	- Not in funiculus - Muscle/striated tissue
*zpg* (*Inx4*)	- Absent	- Absent	- Sometimes overlapping/adjacent to tormogen cells - Localization excludes thecogen cells and OSNs - Labeled cells are mutually confluent
*shakB* (*Inx8*)	- Exclusively neuronal - Heterogenous enrichment across OSN types	- Orco^+^ OSNs - Other antennal cells	N/A

### Innexins likely contribute to forming functional gap junctions in the *Drosophila* antenna

Though no formal gap junction activity has yet been demonstrated for chemosensory sensilla within insect antennae, it is likely that antennal innexin expression maintained into adulthood produces functional innexin channels (innexons). No functions aside from channel formation have been described or attributed to innexin proteins. Despite no primary amino acid sequence homology to the better-described connexin family in chordates (Hua et al. 2003), innexins and connexins are dramatically similar in structure, sharing identical membrane topology and consisting of four transmembrane domains, two extracellular and one intracellular loop, along with intracellular N- and C-terminal tails (Scemes et al. 2009; Pereda 2014). Individual innexons are made from eight innexin subunits, instead of the six subunits of connexons, and assemble into homo- or heteromultimers around a central channel which forms a transmembrane pore (Phelan et al. 1998b, 1998a; Holcroft et al. 2013; Hasegawa and Turnbull 2014; Oshima et al. 2016b, 2016a; Sánchez et al. 2019; Burendei et al. 2020). Examples of other similarities include conserved Cys residues within extracellular loops, where connexins harbor three Cys residues and innexins carry two Cys residues (Scemes et al. 2009). It is therefore most plausible that both abundant transcription and translation of innexin genes in the antenna as we observe produces functional innexons. Innexin presence is unlikely to be left over from development, where innexins and associated gap junctions contribute to mediate development at least in embryogenesis (Stebbings et al. 2002; Bauer et al. 2003, 2004; Ostrowski et al. 2008; Giuliani et al. 2013; Hasegawa and Turnbull 2014; Güiza et al. 2018), especially given that cells in the *Drosophila* antenna are thought not to undergo any turnover or proliferation following metamorphosis and eclosion, with a singular, provisionally reported exception concerning mechanosensory neurons of the Johnston’s organ (Hernandez et al. 2021).

### Neuronal innexin expression and possibility of cross-neuronal junctioning

Bidirectional movement of ions and metabolites can permit neurons to alter membrane potentials of adjoined neurons (Pereda 2014). In the sensilla, grouped neurons exhibit stereotypic close proximity, a large contact surface between neurons, and shared sheathing provided by the thecogen cell (Shanbhag et al. 2000; Ng et al. 2020; Prelic et al. 2022). Our dye confinement observations do not rule out that co-housed neurons may themselves be mutually coupled through gap junctions. In gustatory sensilla of adult worker *Bombus terrestris* bumblebees, whose gustatory sensory neurons are coherently coupled in activity, pharmacological blocking of gap junctions using carbenoxolone abolished characteristic burst firing responses, demonstrating neuron–neuron interactions facilitated by gap junctions (Miriyala et al. 2018). Similarly, in adult *Drosophila* wing chemosensory sensilla, a gap junction–like function of *Inx2* was inferred, wherein genetic knockdown of *Inx2* negatively affected Ca^2+^ wave propagations along the *Drosophila* anterior wing margin nerve in response to presentation of tastant stimuli (Raad and Robichon 2019). Similar parallels exist in visual systems, where gap junctional coupling between cone photoreceptors is found to decrease intrinsic neuron noise, and thus increase sensitivity and fidelity of sensory neuron signals (Bloomfield and Völgyi 2009). Tentatively, a putative neuron–neuron gap junction hypothesis may be in line with recent attempts to model multi-OSN interactions which exclude the participation of support cells, and which suggests that grouped OSNs housed in the same sensillum may electrically interfere or interact with one another to improve identification of concentration ratios in odorant mixtures, and in discriminating odorant mixtures emanating from singular sources (Pannunzi and Nowotny 2021). Gap junctions connecting co-housed OSNs may also be in part responsible for observed electrical (“ephaptic”) inhibition between co-housed OSNs (Su et al. 2012; Zhang et al. 2019). However, electrical interference between neighboring neurons does not necessarily require gap junctions nor conventional forms of neuronal communication, and are thus generally thought unlikely or not to occur on (olfactory) sensory neurons at the sensory periphery to date (Su et al. 2012; Retamal et al. 2017). We consider electrical synapsing at the olfactory receptor level an implausible hypothesis as gap junctions would more likely facilitate activation rather than inhibition of neighboring neurons, as is the case in the gap junction–adjoined neurons in honeybee gustatory sensilla (Miriyala et al. 2018). Other means of sensory input integration exist in compartmentalized sensory neurons which may in part explain these phenomena in the absence of cell–cell communication facilitated by gap junctions (Ng et al. 2020).

However, contrary to this line of thought, we found the shakB protein overtly localizing to a subset of OSNs. A possible explanation of the apparent role of this innexin in OSNs involves their trafficking for localization at the axonal termini of OSNs, where they could form electrical synapses in the central nervous system. However, axonal termini of OSNs appear to be entirely free of electric synapses at the antennal lobe (Yaksi and Wilson 2010; Wilson 2013; Das et al. 2017). In fact, instances of lateral signaling between some OSNs were also recently shown to be entirely *shakB*-independent within the antennal lobe region (Zocchi et al. 2022). This likely excludes the possibility that innexins are trafficked within OSNs to their synaptic termini, and rather implies that any innexin expression in OSNs is likely restricted spatially to the antennal periphery. This inference is supported by observations that OSN responses are coupled with those of antennal glia, as surveyed by live Ca^2+^ imaging of antennal glia (Calvin-Cejudo et al. 2023). Here, OSN activation-dependent coupling in physiological activity between OSNs and glia may be dependent on *shakB* expression in glia, as *shakB* silencing in antennal glia by RNAi—but not silencing of K^+^ channels *Irk1*/*Irk2* or taurine/aspartate transporter *Eaat2*—recapitulates perceptual deficits observed in flies with ablated antennal glia (Calvin-Cejudo et al. 2023). Curiously, the effects in this study were restricted entirely to the antenna, suggesting that glia and OSNs may involve innexons at the antennal level. We have noted a similar interaction previously between thecogen support cells and OSNs, where thecogen cells uptake K^+^ associated with K^+^ extrusion from OSNs responding to odor presentation events (Prelic et al. 2022). However, given that we found no definite glia-specific expression of *shakB*, thecogen-specific expression of innexins, and observed gap junction-permeable dye confinement to neurons alone, we cannot conclude that gap junctions facilitate intercellular connections mutually among neurons, at least at the level of the sensillum.

We also caution that our backfilling experiment is a singular case study, and there remains a possibility that sensillum types other than the sampled ab2 sensillum may harbor gap junctions between neurons and support cells. This is obvious given the great number of chemosensory sensilla located on the third antennal segment. Concomitant increases in intracellular Ca^2+^ in all OSNs and an unknown subset of tormogen cells following odor stimulation have been observed in Ca^2+^ imaging in open antennal preparations (Prelic et al. 2022), which might imply that some sensilla, though not all, exhibit coupling between cell types. However speculative, any identification of a sensillum wherein OSNs directly couple to support cells would thus indicate that a “gap junction heterogeneity” exists between sensilla, further subclassifying an already diverse set of extrasensory sensilla. In such a hypothetical case, the presence or absence of gap junctions could perhaps in part explain different, unaccounted for properties of OSN subclasses such as differences in an OSN’s resting/spontaneous spiking activity or variable sensitization (Halty-deLeon et al. 2021).

Lastly, we note that there exist few comparable investigations concerning the question of OSN-OSN coupling in the olfactory periphery of animals. For instance, an almost total absence of mature OSN-to-OSN–bridging gap junctions was shown in the olfactory epithelium of salamanders, where rare incidences of dye coupling between adjacent OSNs were confounded by inter-neuronal coupling between nascent and immature OSNs (Delay and Dionne 2003). Conversely, mature OSNs harboring gap junctions were demonstrated in mice, wherein removal of gap junctions led to decreased sensitivity as measured by electrophysiological means (Zhang 2010). In light of these efforts, our pilot study unfortunately does not provide a definitive result with regard to *Drosophila* OSNs.

### Heterogeneity in neuronal *shakB* expression

As noted prior, single-cell transcriptomics in the antennae revealed that *shakB* expression occurs in both IR and OR olfactory subsystems. Interestingly, *shakB* itself showed considerable gene expression variability among OSN subclasses: *shakB* was depleted in groups consisting of large olfactory neurons of large basiconic sensilla—those expressing *Or22a* (ab3A neuron), *Or42b* (ab1A neuron), and *Or59b* (ab2A neuron)—as well as in the large neuron of a trichoid sensillum expressing *Or47b* (at4A neuron). For most other olfactory neuron groupings, *shakB* was found significantly enriched relative to other antennal cells, irrespective of olfactory subsystem. We propose that this observation may in part account for differences in neurophysiology of morphologically large and small neurons housed within the same sensillum. One potential explanation may be that smaller neurons may disproportionately harbor hemichannels connecting them to the perineuronal lumen, which may provide a rationale for the ephaptic inhibitory effect observed upon large neuron activation which asymmetrically inhibits activity in the smaller neuron of the sensillum (Zhang et al. 2019). This explanation is rendered cogent given that all inner dendritic and somatic regions of grouped OSNs are ensheathed by processes of the thecogen cell, which is thought to create a sealed perineuronal lumen for the neurons it envelops (Prelic et al. 2022).

### Gap junctions may interconnect non-neuronal classes of support and epithelial cells

In our study, we observed most antennal innexins displaying non-neuronal localizations. Given that gap junction proteins are very highly expressed in mature antennae, gap junction connections may therefore interconnect support and epithelial cells among each other. Support cells are known to be coupled to each other in other sensory systems featuring regulated lymph compartments such as the mammalian cochlea (Zhu et al. 2013). Gap junctions in sensilla may facilitate metabolic coupling between neighboring cells, such as tormogen and trichogen cells, which are rich in ultrastructural features indicating active metabolism (Gnatzy and Weber 1978; Steinbrecht 1996; Willingham and Keil 2004; Prelic et al. 2022). Tormogen and trichogen cells are also both closely apposed with each other and epithelial cells (Nava Gonzales et al. 2021), and conjoined structurally with septate junctions that are commonly observed in tandem with gap junctions in invertebrates (Keil and Steinbrecht 1987; Steinbrecht 1996). In such a case, gap junctions could serve to exchange sensory system–relevant molecules such as cyclic nucleotides, by-products of lymph maintenance such as digested catabolites, or H^+^ in a pH-buffering capacity (Bauer et al. 2005). More so, any serial arrangement of gap junctions of supporting cells could structurally serve as the basis for recycling lymphatic K^+^ that passes through OSNs during odor transduction, as occurs in the support cells of the cochlear system (Kikuchi et al. 1995). This arrangement within sensilla would be somewhat analogous to the interconnection of satellite glial cells, which are interconnected in vivo to compartmentalize the electrophysiological space of tripartite synapses (Retamal et al. 2017). In insect sensilla in particular, it has been previously reported that high solute (especially K^+^) concentrations in the lymph must undergo strict regulation to ensure appropriate neuron excitability as is crucial for other sensory systems (Corey and Hudspeth 1979; Thurm and Kuppers 1980; Steinbrecht 1989). This hypothesis is in line with reports of K^+^ uptake during odor stimulation by the OSN-adjacent thecogen cells, which are suggested to buffer neuronal K^+^ release (Prelic et al. 2022). Lastly, direct support cell coupling by way of gap junctions may also act as principal pathways to support synchronized activity and contribute to sensory, physiological, or circadian rhythms as evident in fly olfaction (Tanoue et al. 2004), or in other cellular rhythm generating networks in insects (Anava et al. 2009).

### Hemichannels, rather than gap junctions, may be harbored by non-neuronal cells within sensillum architecture

We speculate that if support cells are not electrically coupled with each other, they may instead express innexins which assemble into hemichannels. Innexins are also known to form hemichannels (Bao et al. 2007; Skerrett and Williams 2017), and as such may connect support cells to the sensillum lymph or the small perineuronal lymph compartment sometimes observed between neuron and its sheathing, glia-like thecogen cell (Steinbrecht 1980; Keil and Steinbrecht 1983, 1987; De Kramer 1985; Shanbhag et al. 2000). These hemichannels may ostensibly aid in the release or uptake of small (accessory) molecules that participate in the sensory lymph, such as in the homeostatic maintenance of ionic concentrations of the sensillum lymph (Kofuji and Newman 2004). Alternatively, hemichannels may play a role in the release of local, paracrine signals detectable by other cells of the sensillum (Wang et al. 2013; Orellana and Stehberg 2014). In such a signaling capacity, molecules like glutamate, GABA, and ATP have been reported to act in sensory systems and synchronize activity between cell types (Retamal et al. 2017; Jaeger et al. 2018). For instance, such signal waves may induce changes in intracellular Na^+^ or Ca^2+^ concentrations in adjacent cells (Wang et al. 2013; Retamal et al. 2017), which may in turn potentiate or affect the functional unit of the sensillum or its OSN(s). Given recent findings that cytosolic Ca^2+^ increases acutely during odor detection in tormogen support cells (Prelic et al. 2022), it is tempting to speculate that solute or signaling molecule release may be induced or mediated by Ca^2+^ influx in support cells, and modulate neuron activity via hemichannels akin to gliotransmission (Retamal et al. 2017). ATP and its metabolites are known to bind to purinergic receptors; in such a capacity, they have been shown to function in cochlear support cells by reducing sensory neuron excitability though binding to autoreceptors present on support cells, to induce K^+^ release and subsequent support cell crenation (Babola et al. 2020). Similarly, glial support cells in the chemosensory system of the invertebrate model *C. elegans* detect and release GABA to inhibit neighboring neuronal activity to promote adaptation to noxious stimuli (Duan et al. 2020), while tuning neuronal activity and consequent behavior by active and precise engulfment of neuronal processes as means of sensory modulation (Raiders et al. 2021). Similar mechanisms dependent on release or uptake via hemichannels may exist in the *Drosophila* antenna. This is presumed as activity-coupling mechanisms between cells of the sensillum have been scarcely investigated (Prelic et al. 2022).

### Putative regulation of innexon channel function in context of chemosensory sensilla

Lastly, it is important to underscore that the opening and closing of hemichannels and gap junctions can be triggered by multiple phenomena, many of which continually occur at the *Drosophila* olfactory periphery. There exist precedents that dynamic regulation of electrical junctions can occur in a fashion dependent on sensory modality, such as illumination or circadian rhythm in the visual system (Bloomfield and Völgyi 2009). Though innexons are canonically non-selective, evidence exists that some animal gap junction pores exhibit ion selectivity (Beblo and Veenstra 1997; Wang and Veenstra 1997; Suchyna et al. 1999), and where additional, known properties of gap junctions such as rectification and regulation by post-translational modification or via chemical or voltage gating may too be relevant to consider (Nielsen et al. 2012). For instance, pore size and resultant permeability have been shown to depend on both cytoplasmic and extracellular ionic concentrations (Nielsen et al. 2012; Skerrett and Williams 2017). Here, cytosolic Ca^2+^ can uncouple cells partnered by gap junctions (Baux et al. 1978); Ca^2+^ is known to rapidly increase following odor stimulation in OSNs, and has recently been shown to increase in a subset of tormogen cells in response to OSN activation as well (Prelic et al. 2022). Innexin channel permeability is also affected by transmembrane and transjunctional voltage, such that gap junctions open in a manner dependent on depolarization (Bao et al. 2007; Skerrett and Williams 2017). All such modes of gap junction regulation are readily present within the insulated, closed compartments of insect sensilla. The presence and putative function of antennal innexins must therefore consider innexon channel regulation on the one hand, and the organismal role of the antenna as an olfactory organ, which performs continuous detection of broad ranges of odor inputs appearing in a turbulent odorscape. We therefore predict that innexon channel regulation may be naturally suited to act in sensilla given the dynamics of odor sensing and the short temporal scales involved in high-fidelity detection of odors.

### Limitations and future directions for antennal gap junction research

Based on the apparent antennal abundance of innexins, it is clear that the contributions of innexon channels to the process of odor detection requires further study. One major obstacle to further understanding on this front is that innexins do not seem to be overtly sensillum subtype- or neuron class-specific, which creates an exploratory barrier for targeting sensilla in defining or pinpointing a locus for testing gap junction involvement. Another barrier is that most antennal innexins, and presumably gap junctions, are not neuronal. This presents technical challenges to access, physiologically characterize or probe non-neuronal cells during chemosensing, which do not feature overt responses to chemical stimuli such as those characteristic of depolarizing sensory neurons. This problem ultimately makes it difficult to test hypotheses and predictions in experiments involving mutationally knocking out, overexpressing, pharmacologically blocking, or genetically silencing innexins for functional investigation in underexplored cell types such as those of support cells. For instance, it is unknown what specific features of an olfactory response would be diagnostic of gap junction-dependent activity. And, if any, how could they be feasibly probed in non-neuronal cells? Would measurements at the periphery or antennal lobe be more suitable loci for detecting impairments in odor signal transmission as a result of experimentally induced impairments in gap junctional transmission of signals at the funiculus? Many such challenges and technical considerations exist.

Despite limitations in accessibility of antennal sensilla for dye coupling or gap junction blocking experiments, we suggest a more systematic investigation of innexins through use of gene silencing in tandem with available single sensillum recording techniques. For instance, we expect cell type-targeted genetic knockdowns of antennal innexins, followed by electrophysiological measurements of odor responses, to be viable avenues for discovery of specific gap junction activity. Foremost, we expect electrophysiological techniques to have sufficient temporal discrimination to resolve fast, short-lived or electrical effects which may reveal contributions of gap junctions within a sensillum responding to specific odors. Second, single sensillum recordings have an advantage of recording electrical activity within the confines of a single sensillum, thus allowing for favorable, sensitive, and unimpeded measurement of activity which may be restricted to singular sensilla. Additionally, genetic silencing of innexin expression may instead also be substituted by probing sensilla of innexin mutant fly lines, some of which are currently available (e.g., *ogre*, *shakB* mutant lines). Alternatively, given our broad detection of innexins across many antennal cells and cell types, real-time Ca^2+^ imaging of generic olfactory responses in open antennal preparations in the presence and absence of pharmacological compounds blocking innexin gap junctions may also be a fruitful direction for future research. Compounds such as carbenoxolone, brilliant blue G, ATP, arachidonic acid, and probenecid have been identified and successfully employed to selectively block gap junctions in previous research on invertebrates (Bruzzone et al. 2005; Bao et al. 2007; Silverman et al. 2008; Qiu and Dahl 2009; Dahl and Muller 2014; Sangaletti et al. 2014). However, both electrophysiology and functional imaging feature an important caveat that the precise ultrastructural location within the sensillum compartment, and the determination specific cellular interconnectivity, will not be derivable.

## Conclusion

To our knowledge, this study presents a first investigation into the localization and potential function of gap junctions in the periphery of the olfactory system of *Drosophila*. Further studies are required to determine the ultrastructural localization of potential gap junctions or hemichannels, and to elucidate any potential roles they may exhibit in adjoining various cells and compartments present. Ultimately, it will be crucial to understand how such multicellular sensory systems coordinate the ever-present tasks of detecting and processing odors and lymphatic compartments, and the extent to which support cells functionally assist sensory neurons.

## Supplementary information

Below is the link to the electronic supplementary material.

Supplementary file1 (PDF 10953 KB)

## Data Availability

Data used for this study is made available in the data repository EDMOND at the following link: https://doi.org/10.17617/3.OGRUNX
