# Supplementary material for: Innexin expression and localization in the Drosophila antenna indicate gap junction or hemichannel involvement in antennal chemosensory sensilla
Source: Cell Tissue Res. 2024 Aug 23;398(1):35–62. doi: 10.1007/s00441-024-03909-3 (PMC11424723; doi:10.1007/s00441-024-03909-3)
Supplement: Supplementary file 1 — Supplementary file1 (PDF 10953 KB) [file 441_2024_3909_MOESM1_ESM.pdf]

# Supplementary Figure S1

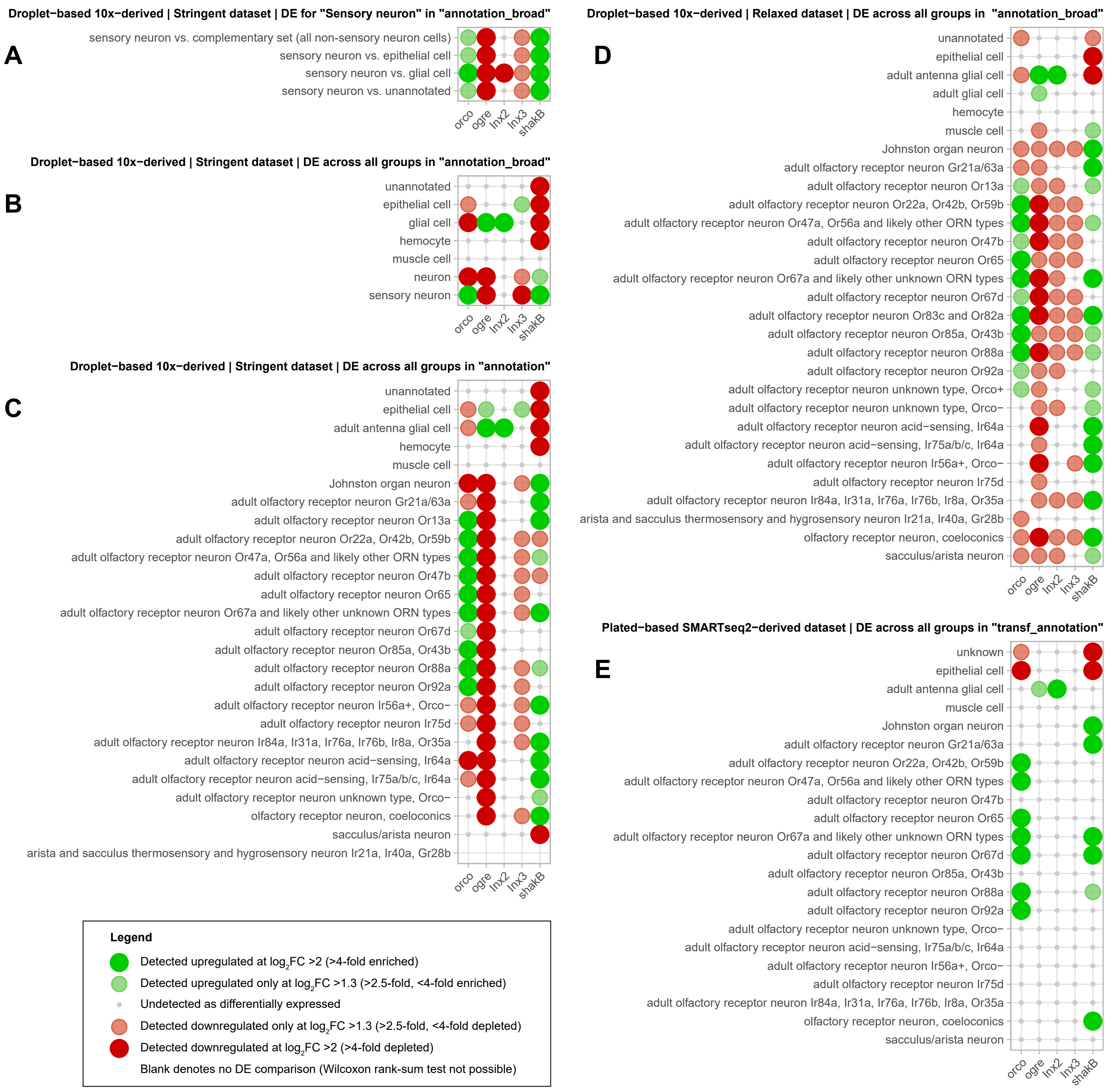

**Supplementary Figure S1. Extended differential expression analysis.** Differential up/downregulation results are consistent across various modes of comparison, various single cell RNA-seq dataset types and single cell capture methods. Here, statistically significant differential expression among antennal innexins (*ogre*, *Inx2*, *Inx3*, *shakB*) and *Orco* are reported across various cell grouping schemes and scRNA-seq datasets, with a more varied and finer-grained approach. (**A**) For the 10x, stringent antennal dataset (Fly Cell Atlas), differential expression analysis is performed focusing on the "Sensory neuron" subset of cells in the "annotation_broad" classification annotation; all tested comparisons are explicated. *shakB* is enriched sensory neurons, while *ogre*, *Inx2* and *Inx3* are relatively depleted relative to epithelial, glial and unannotated cell subsets. Differential expression analysis is also performed for the 10x stringent antennal dataset based on the broad cell type-specifying "annotation_broad" annotation (**B**) and for the narrow cell type-specifying "annotation" annotation (**C**). Equivalent differential expression analysis was also performed on the relaxed antennal dataset that does not feature 'stringent' preprocessing steps, which aims to clean up data but may introduce biases (e.g. "overcorrection or removal of non doublet cells" (Li et al., 2022a)) (**D**) and an antennal scRNA-seq dataset of different cell capture methodology, derived from the plate-based SMART-seq2 cell capture approach (in contrast to the 10x droplet-based approach) (**E**), which has a benefit of deeper sequencing per cell (higher gene detection rate). Unlike in Figure 2d, the plot reports differentially regulated expression categorically rather than as continuous data: only significantly differentially expressed genes are reported, in small translucent circles (when log2foldchange > 1.3 but < 2.0) or large opaque circles (when log2foldchange > 2.0). Red and green indicate statistically significant downregulation and upregulation, respectively. Blank comparisons denote statistical comparison (Wilcoxon rank-sum test) was not possible for the given dataset/cell grouping.

# Supplementary Figure S2

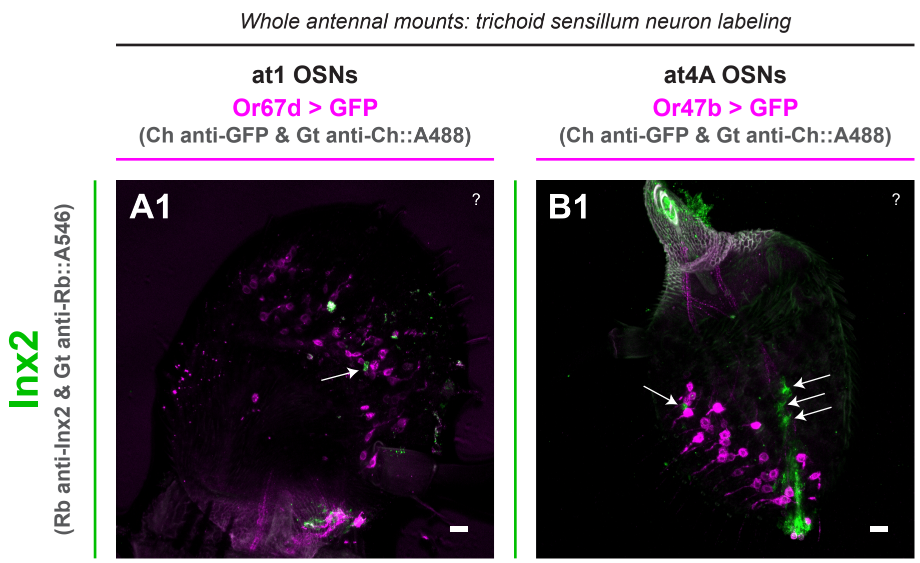

**Supplementary Figure S2. Whole antennal mounts labeling Inx2 and trichoid neurons.** Based on prior whole antennal immunofluorescence imaging, Inx2 staining localized to distal portions of the antenna. We performed a repeated Inx2 staining experiment in animals with Gal4-driven fluorescent labeling in at1 (Or67d$^{+}$) (**A1**) and at4A (Or47b$^{+}$) (**B1**) neurons to determine degree or potential extent of Inx2 colocalization with these neurons that are enriched in distal areas of the funiculus. Arrows indicate cell-like structures. The staining seems to be dependent on degree of tissue accessibility, i.e. if the cuticle was nicked during preparation to allow for antibody permeability into the antennal tissue. Ch: chicken. Rb: rabbit. Gt: goat. All scale bars: 10 µm.

# Supplementary Figure S3

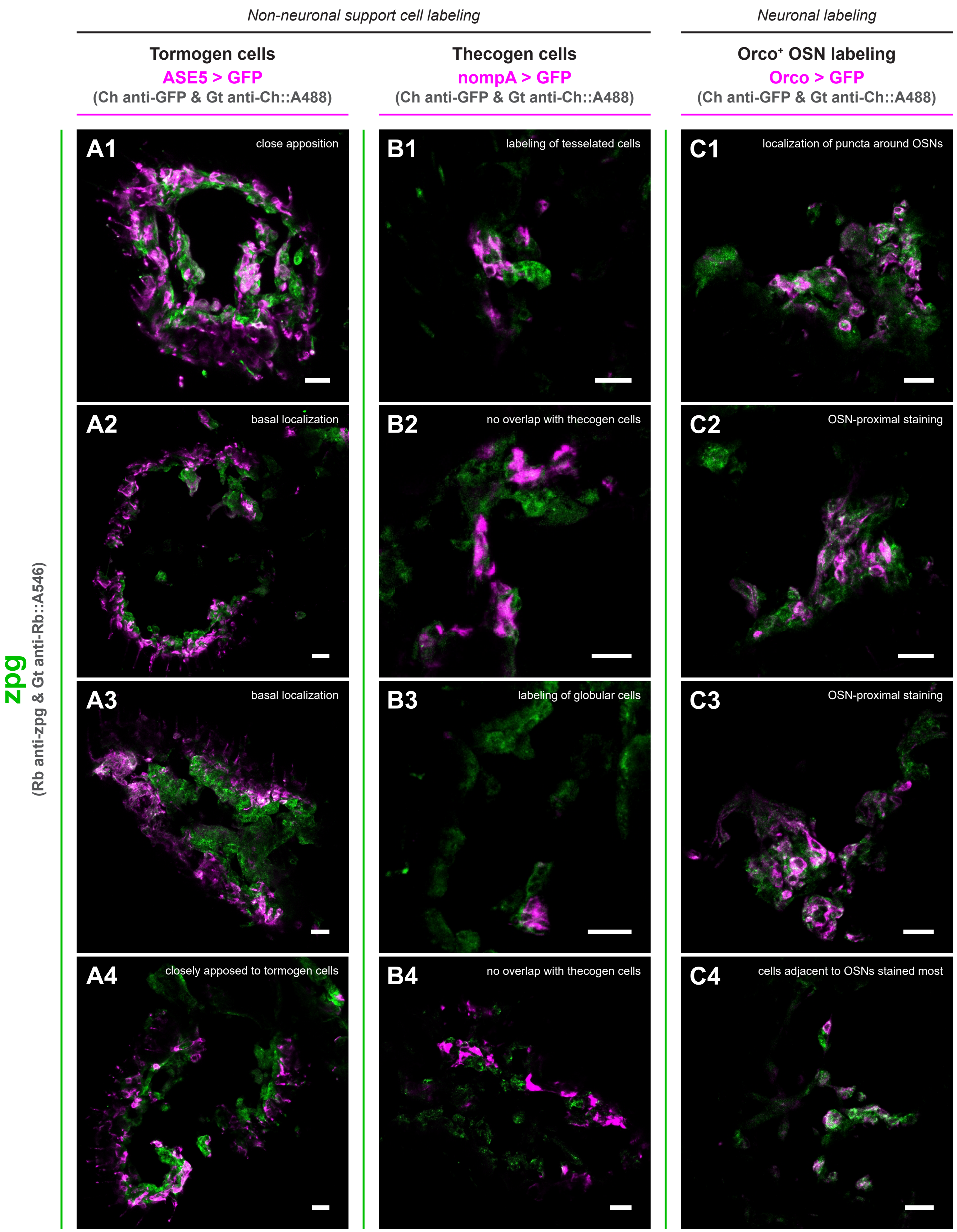

**Supplementary Figure S3. zpg immunofluorescence staining in cryosectioned antennal tissue with support cell and OSN counterstaining.** As a colocalization study, zpg immunostaining was performed in sections of antennae expressing Gal4-driven fluorescent labeling of tormogen socket cells (**A**), thecogen sheath cells (**B**), and Orco$^{+}$ OSNs (**C**). For each counterstaining (A-C), four exemplars (1-4) are provided. Observations of zpg protein localization are summarized for each panel. Ch: chicken. Rb: rabbit. Gt: goat. All scale bars: 10 μm.

# Supplementary Figure S4

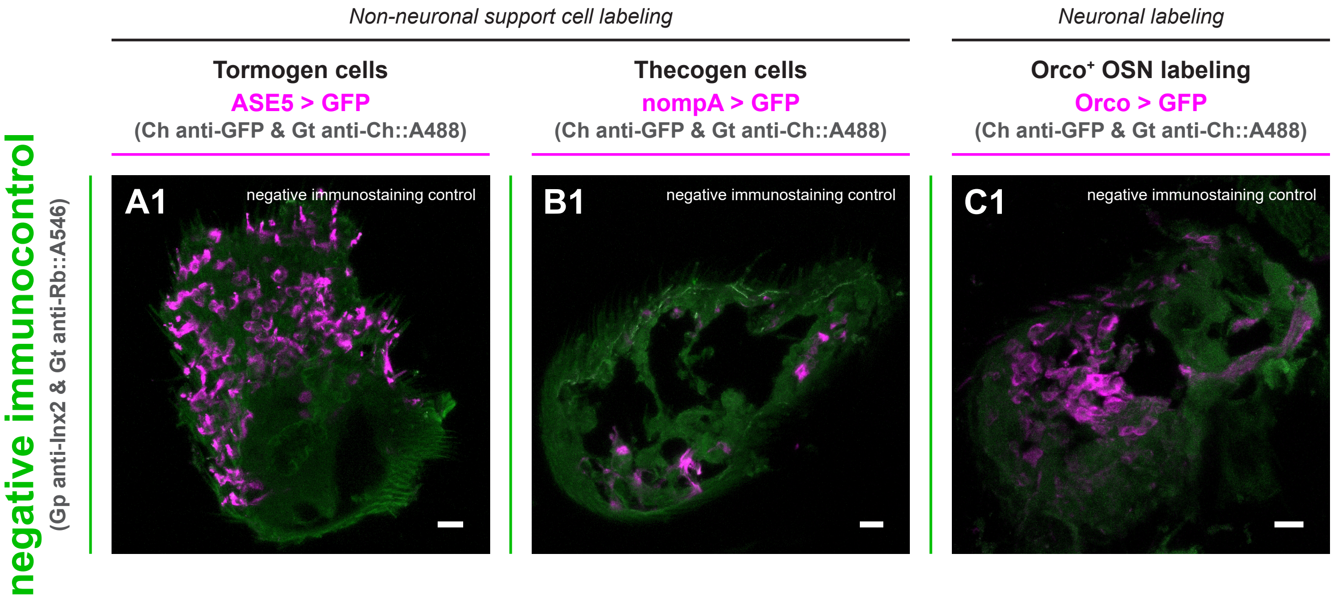

**Supplementary Figure S4. Negative immunostaining control with mismatched antibodies to determine background staining levels.** Secondary antibodies specific to rabbit epitope were employed with goat anti-Inx2 primary antibodies as a negative control in sections of antennae expressing Gal4-driven fluorescent labeling of tormogen socket cells (**A**), thecogen sheath cells (**B**), and Orco$^{+}$ OSNs (**C**). The stainings serve as noise baselines for judging positive staining in other immunofluorescence experiments. Ch: chicken. Rb: rabbit. Gp: guinea pig. All scale bars: 10 µm.
